# Oligodendrocyte dysfunction contributes to motor deficits and Purkinje cell axonopathy in spinocerebellar ataxia type 1

**DOI:** 10.1172/JCI195723

**Published:** 2026-05-07

**Authors:** Changwoo Lee, Rosalie M. Grijalva, Leon Tejwani, Eunwoo Bae, Alison Chase, Hannah Ro, Hannah Kim, Victor Olmos, James P. Orengo, Janghoo Lim

**Affiliations:** 1 Interdepartmental Neuroscience Program and; 2 Department of Genetics, Yale School of Medicine, New Haven, Connecticut, USA.; 3 Yale College, New Haven, Connecticut, USA.; 4 Departments of Neurology and Neuroscience, Baylor College of Medicine, Houston, Texas, USA.; 5 Department of Neuroscience;; 6 Program in Cellular Neuroscience, Neurodegeneration and Repair;; 7 Yale Stem Cell Center; and; 8 Wu Tsai Institute, Yale School of Medicine, New Haven, Connecticut, USA.

**Keywords:** Genetics, Neuroscience, Neurodegeneration, Neurological disorders

## Abstract

Spinocerebellar ataxia type 1 (SCA1) is a neurodegenerative disease marked by progressive motor deficits and Purkinje cell (PC) degeneration, driven by polyglutamine expansion in ataxin-1. While oligodendroglial dysfunction precedes PC loss, its direct contribution toward SCA1 pathogenesis remains unclear. Here, using an oligodendroglia-specific SCA1 conditional knockin mouse model, we demonstrate that mutant ataxin-1 in oligodendrocytes is sufficient to drive aspects of SCA1-related pathology, including dysregulated myelination, PC axonal shrinkage, and torpedo formation, ultimately impairing motor coordination. Transcriptomic analysis uncovers cerebellar oligodendrocyte subtypes with distinct gene expression signatures and aberrant abundance that contribute to demyelination. This, compounded by a progressive decline in the neuroprotective functions of a cerebellum-specific oligodendrocyte subtype, establishes a critical link between demyelination, axo-myelinic dysfunction, and axonal pathology in SCA1. Upstream transcriptional regulator analysis in oligodendroglia identifies transcription factor 7-like 2 (TCF7L2) and huntingtin (HTT) as key mediators of oligodendroglial dysfunction in SCA1, suggesting shared pathogenic mechanisms with other polyglutamine diseases. Collectively, these findings establish oligodendroglia as key mediators of SCA1 pathogenesis and underscore their critical role in preserving PC axonal integrity.

## Abstract 2

This study provides mechanistic insights into the early, cell-autonomous and non-cell-autonomous contribution of oligodendrocytes to spinocerebellar ataxia type 1 (SCA1), a progressive neurodegenerative disorder caused by polyglutamine-expanded ataxin-1

## Graphical abstract

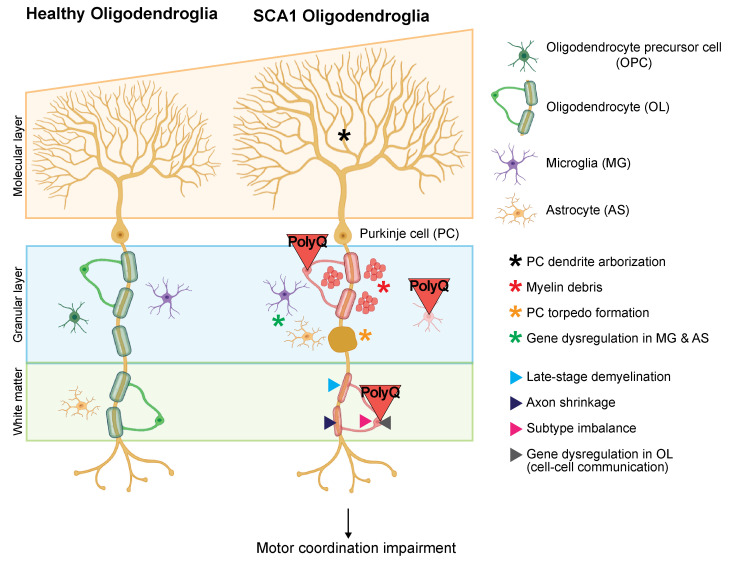

## Introduction

Spinocerebellar ataxia type 1 (SCA1) is an autosomal dominant neurodegenerative disorder caused by a CAG repeat expansion in the ataxin-1 (*ATXN1*) gene, producing polyglutamine-expanded (polyQ-expanded) ataxin-1 (1, 2). This mutation primarily affects the cerebellum, triggering progressive motor deficits and Purkinje cell (PC) degeneration (1). SCA1 belongs to a family of polyQ diseases, including Huntington’s disease (HD), spinal and bulbar muscular atrophy, dentatorubral-pallidoluysian atrophy, and other SCAs (SCA2, SCA3, SCA6, SCA7, and SCA17) (1). While these disorders share common motor impairments, they exhibit distinct patterns of neuronal vulnerability and regional central nervous system (CNS) involvement (1, 3).

Emerging evidence suggests that white matter abnormalities play a critical role in SCA1 pathology (4, 5). Magnetic resonance imaging studies have revealed myelination abnormalities in patients with SCA1, akin to those seen in SCA2, SCA3, SCA7, and HD (6–13). Given that oligodendrocytes (OLs) are essential for myelination, axonal maintenance, and neuronal signaling, OL dysfunction may be a key driver of the motor symptoms observed across polyQ diseases (14, 15). These findings identify OLs as a highly vulnerable population in polyQ toxicity, with potential implications for disease progression.

Beyond its established role in SCA1, *ATXN1* mutations are increasingly linked to a spectrum of neurodegenerative conditions. While uninterrupted CAG repeat expansions of 39 or more (up to 83 repeats) cause fully penetrant SCA1 (16), intermediate expansions (32–39 repeats) are associated with an increased risk of sporadic amyotrophic lateral sclerosis (ALS) (17, 18). Intriguingly, intronic mutations that reduce *ATXN1* expression have been implicated in Alzheimer’s disease (AD) (19, 20) and multiple sclerosis (MS) (21, 22). These diseases are also characterized by myelination deficits and OL dysfunction (23–28). These shared disease features suggest a broader role for ataxin-1 in oligodendroglia function beyond SCA1. To model the disease, the *Atxn1^78Q/+^* knockin mouse was generated; however, even in the homozygous state, it exhibited only minimal motor behavior phenotypes (29). In contrast, the *Atxn1^154Q/+^* knockin model (hereafter referred to as SCA1-KI) develops robust pathology, characterized by progressive motor coordination defects beginning at approximately 5 weeks, weight loss starting around 8 weeks, PC loss emerging near 34 weeks, and premature death by 45 weeks (30). While reproducing human-relevant phenotypes in mice often requires longer CAG repeat expansions, in part due to their shorter lifespan (29–32), this model provides a valuable tool for investigating SCA1 pathogenesis. Our previous work, using single-nucleus RNA sequencing (snRNA-seq) on postmortem cerebellar tissue from patients with SCA1 and the SCA1-KI mouse model, uncovered early and significant dysregulation in oligodendrocyte progenitor cells (OPCs) and OLs that precedes PC degeneration (33). However, the precise cellular and molecular mechanisms through which oligodendroglia may contribute to SCA1 pathogenesis remain unclear.

To directly investigate the role of oligodendroglia in SCA1, we developed an oligodendroglia-specific SCA1 conditional knockin (OL-SCA1-cKI) mouse model, selectively expressing mutant ataxin-1 in OL lineage cells. Our studies revealed that dysfunction of cerebellum-specific OL is sufficient to drive SCA1-related pathology and motor deficits, independent of neuronal degeneration. We demonstrate that mutant ataxin-1 expression within oligodendroglia induces hallmark SCA1 motor impairments, progressive myelin dysregulation, and PC axonal shrinkage with torpedo formation. Using snRNA-seq, we reveal OL subtype imbalances and transcriptomic disruptions that impair neuron-OL communication, identifying dysregulated pathways mediated by transcription factor 7-like 2 (TCF7L2) and huntingtin (HTT), which further suggest shared molecular mechanisms across SCA1 and other polyQ disorders. Collectively, our findings establish OL dysfunction as both a direct, cell-autonomous driver of SCA1 pathology and an indirect, non-cell-autonomous contributor to PC axonal integrity. This work uncovers a previously underrecognized glial contribution to cerebellar neurodegeneration and presents potential therapeutic targets for polyQ-driven cerebellar disorders.

## Results

### Oligodendroglial mutant ataxin-1 expression is sufficient to drive SCA1-related motor phenotypes.

**Figure 1:**
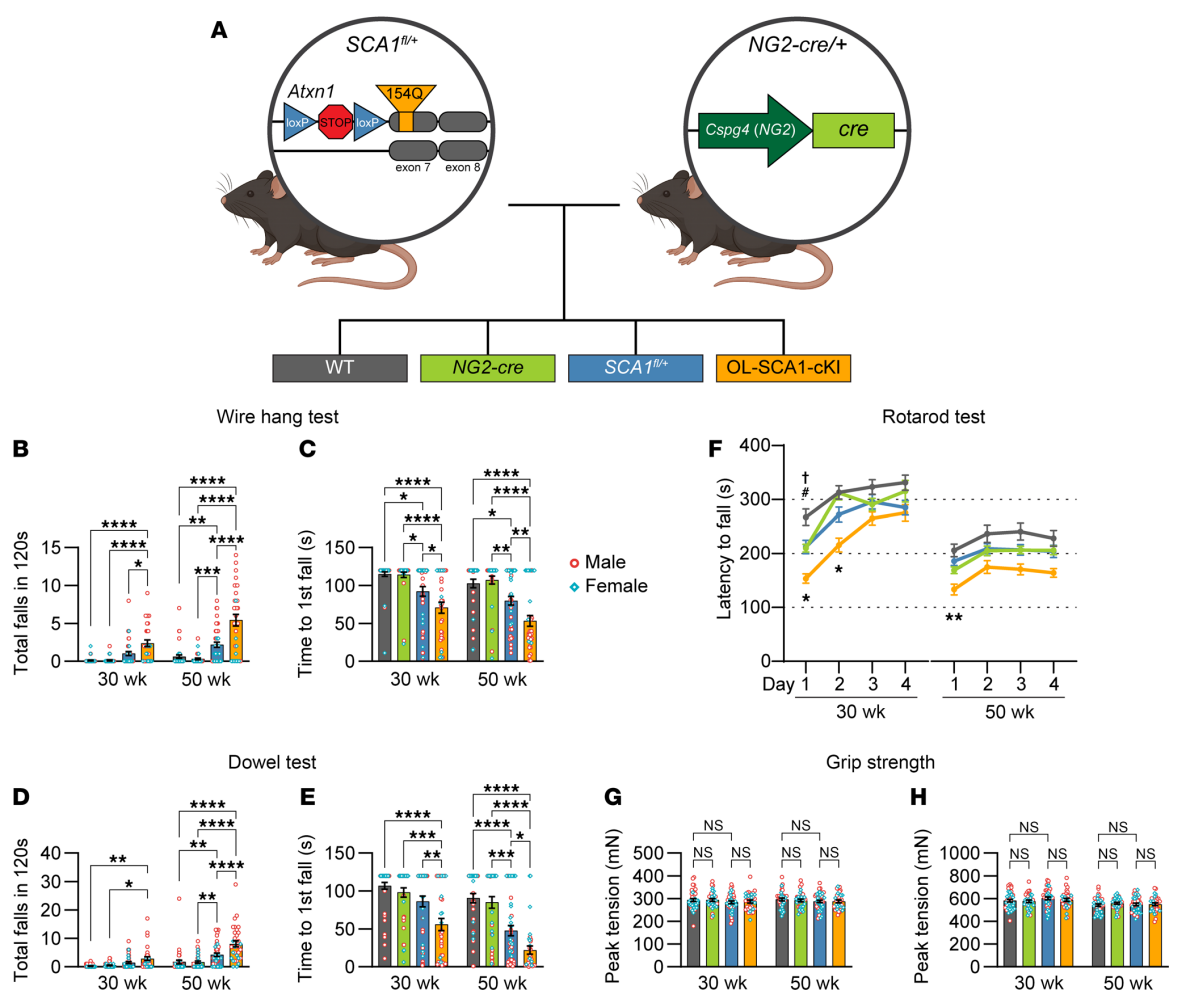
Mutant ataxin-1 expression in oligodendroglia induces SCA1-related motor deficits. (**A**) Breeding schematic depicting the 4 genotypes: WT, NG2-cre, SCA1^fl/+^, and OL-SCA1-cKI (SCA1^fl/+^; NG2-cre). (**B**–**H**) Motor coordination performance assessed in OL-SCA1-cKI and control mice at 30 and 50 weeks of age using wire hang tests (**B** and **C**), dowel rod tests (**D** and **E**), rotarod tests (**F**), and grip strength tests (**G** and **H**). The number of animals (M: male, F: female) is as follows. At 30 weeks of age: WT (21M, 20F), NG2-cre (17M, 19F), SCA1^fl/+^ (23M, 18F), and OL-SCA1-cKI (23M, 11F). At 50 weeks: WT (20M 20F), NG2-cre (17M, 19F), SCA1^fl/+^ (23M, 20F), and OL-SCA1-cKI (22M 11F). Data represent mean ± SEM (**B**–**E**, **G**, and **H**), or weighted mean ± SEM, equally representing male and female mice (**F**). Two-way ANOVA with Tukey’s test: *P < 0.05, **P < 0.01, ***P < 0.001, ****P < 0.0001; all other comparisons are nonsignificant. (**F**) Additional annotations: ^†^*P* < 0.05 (WT vs. NG2-cre); ^#^*P* < 0.05 (WT vs. SCA1^fl/+^); *P < 0.05, **P < 0.01 (SCA1^fl/+^ vs. SCA1^fl/+^; NG2-cre).

To directly assess the role of polyQ-expanded ataxin-1 in oligodendroglia and its contribution to SCA1-related phenotypes, we generated an OL-SCA1-cKI mouse model. These mice were developed by crossing SCA1 conditional KI (*SCA1^fl/+^*) mice (34) and *Cspg4 (NG2)-cre* (*NG2-cre^/+^*) transgenic mice (35, 36), yielding 4 genotypes: wild-type (WT), *NG2-cre*, *SCA1^fl/+^*, and *SCA1^fl/+^ NG2-cre* (Figure 1A). In OL-SCA1-cKI (*SCA1^fl/+^* *NG2-cre*) mice, Cre recombinase is expressed in OPCs under the control of the *Cspg4* (*NG2*) promoter, excising stop codons flanked by *LoxP* sites and enabling expression of the mutant *Atxn1^154CAG^* allele throughout the oligodendroglial lineage, including mature OLs. In the absence of Cre recombination, the mutant *Atxn1* allele in *SCA1^fl/+^* mice remains inactive and silenced, resulting in expression from only the single endogenous WT *Atxn1* allele and creating a heterozygous *Atxn1* background. To confirm oligodendroglia-specific Cre recombination, we crossed *NG2-cre* mice with Ai9 Cre-reporter mice, which express RFP in cells undergoing Cre-mediated recombination (37). IHC of cerebellar tissue revealed robust RFP expression in SRY-box transcription factor 10–positive (Sox10^+^) pan-oligodendroglia and myelin-rich white matter, with minimal expression in nuclear factor 1 A–positive (Nf1a^+^) astrocytes, calbindin–positive (Calb1^+^) PCs, or ionized calcium-binding adapter molecule 1–positive (Iba1^+^) microglia (Supplemental Figure 1, A and B; supplemental material available online with this article; https://doi.org/10.1172/JCI195723DS1). Furthermore, RFP expression was sustained throughout differentiation, colocalizing with stage-specific markers including Pdgfra (OPCs), Tcf7l2 (premyelinating OLs), and CC1 (mature OLs) (38) (Supplemental Figure 1, C–E). This confirmed the successful generation of an oligodendroglia-specific conditional mouse model expressing mutant ataxin-1 [154Q], providing a system to dissect the role of oligodendroglial dysfunction in SCA1 pathogenesis.

We next sought to determine whether oligodendroglial expression of mutant ataxin-1 alone is sufficient to induce hallmark SCA1 phenotypes, including weight loss, reduced lifespan, and progressive motor impairments (30). To account for potential sex differences, we calculated weighted means and SEM for both male and female mice. Surprisingly, mice carrying the *SCA1^fl/+^* allele (both *SCA1^fl/+^* and OL-SCA1-cKI animals), which are heterozygous for *Atxn1* throughout the body, including most cells in the brain, exhibited increased body weight compared with WT and *NG2-cre* controls. No significant difference was observed between *SCA1^fl/+^* and OL-SCA1-cKI mice (Supplemental Figure 2A). Additionally, survival rates remained unchanged across all genotypes (Supplemental Figure 2B). To evaluate motor coordination and function over time, we performed wire hang (39), dowel rod (39, 40), rotarod (41), and grip strength (42) tests at 30 and 50 weeks of age (Figure 1, B–H, and Supplemental Figure 2, C–F). *SCA1^fl/+^* mice exhibited impaired motor performance compared with WT controls (Figure 1, B–F). This phenotype may reflect both the 50% reduction of WT ataxin-1, which may contribute to motor deficits, given the motor impairment observed in full knockout mice (41, 43), and the increased body weight of *SCA1^fl/+^* mice. To account for these baseline variables, *SCA1^fl/+^* mice were used as the primary comparison group for OL-SCA1-cKI mice in subsequent analyses, allowing us to isolate the effect of mutant ataxin-1 in oligodendroglia. Notably, OL-SCA1-cKI mice performed significantly worse than *SCA1^fl/+^* mice, showing progressive motor deficits across multiple behavioral tasks (Figure 1, B–F). Importantly, grip strength remained unaffected across all genotypes (Figure 1, G and H), indicating that the motor impairments in OL-SCA1-cKI mice arise from CNS dysfunction rather than peripheral muscle weakness. Together, these findings demonstrate that mutant ataxin-1 expression in oligodendroglia alone can induce SCA1-related motor deficits.

### Myelin dysregulation and PC axon shrinkage in cerebellum white matter of OL-SCA1-cKI mice.

**Figure 2:**
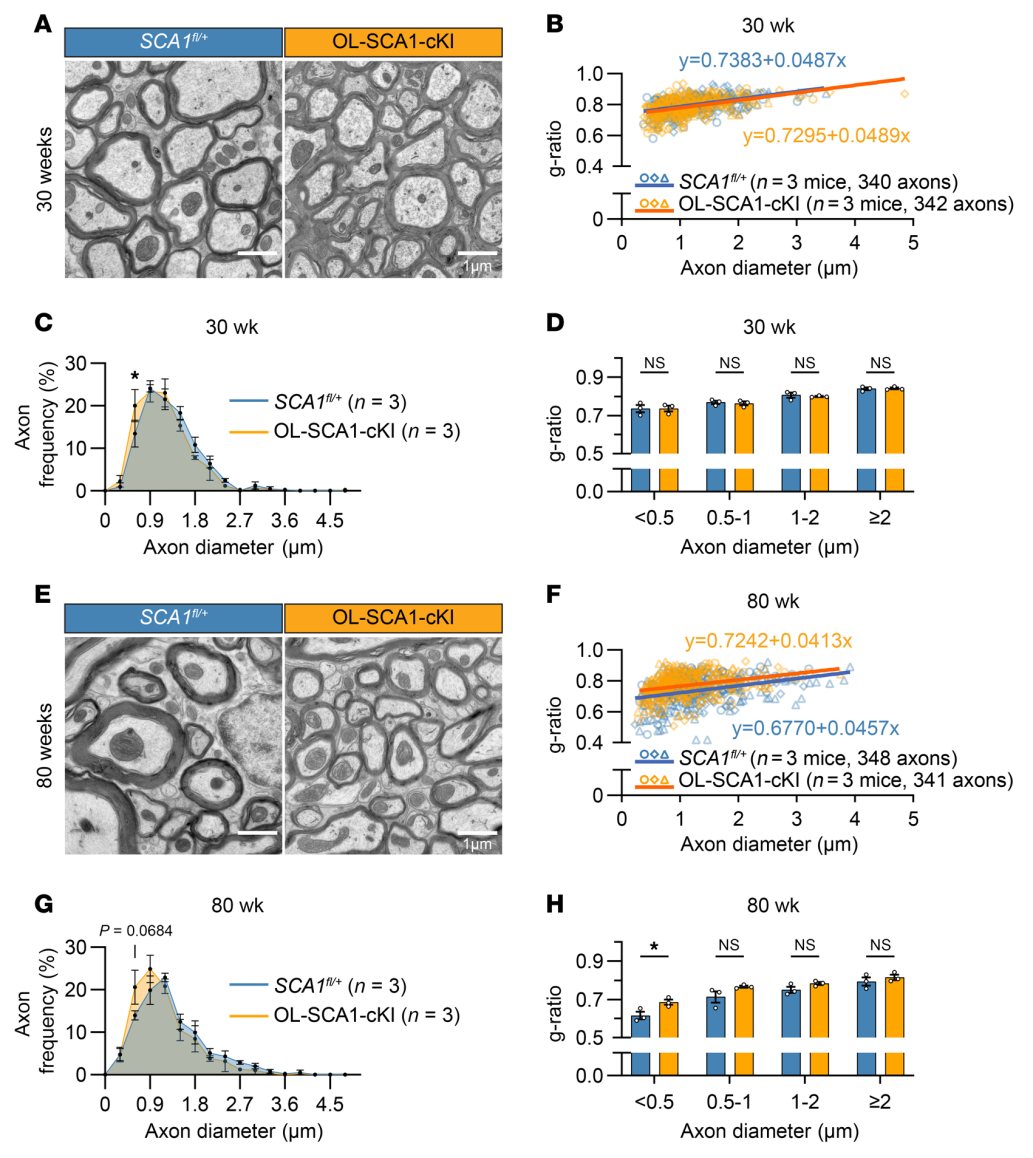
Myelin dysregulation and PC axon shrinkage in OL-SCA1-cKI mice. (**A** and **E**) Representative TEM images of PC axons in cerebellar white matter from SCA1^fl/+^ and OL-SCA1-cKI mice at 30 (**A**) and 80 weeks (**E**). Scale bar: 1 μm. (**B** and **F**) Scatterplots with linear regression analyses of g-ratio versus axon diameter of individual axons at 30 (**B**) and 80 weeks (**F**). *n* = 3 mice per genotype and time point. Axons from individual animals are denoted by distinct symbols (circle, diamond, triangle). (**C** and **G**) Frequency distribution plots of axon diameter at 30 (**C**) and 80 weeks (**G**), demonstrating a shift toward smaller axon calibers (shrinkage) in OL-SCA1-cKI mice starting at 30 weeks. (**D** and **H**) Quantifications of g-ratios by axon diameter at 30 (**D**) and 80 weeks (**H**), revealing significantly thinner myelin (higher g-ratio) in small-caliber axons (<0.5 μm) at 80 weeks. Data represent mean ± SEM with *n* = 3 mice per genotype. Statistical significance was determined using 2-way ANOVA with Šídák’s test (**C**, **D**, **G**, and **H**): *P < 0.05.

The myelination of PC axons, the sole output of the cerebellum and the primary degenerating cells in SCA1, is critical for precise synaptic transmission onto deep cerebellar nuclei neurons (44). To determine how mutant ataxin-1 expression in oligodendroglia contributes to myelin dysregulation and potentially drives motor deficits observed in OL-SCA1-cKI mice, we performed transmission electron microscopy (TEM) on the cerebellar white matter, which predominantly contains myelinated distal PC axons (Figure 2). *SCA1^fl/+^* control and OL-SCA1-cKI mice were analyzed at 30 and 80 weeks of age to assess potential age-dependent changes. At 30 weeks (Figure 2, A–D), OL-SCA1-cKI mice showed a shift toward smaller axon calibers compared with controls, suggesting PC axon shrinkage (Figure 2C). Because thicker axons have relatively higher g-ratio (Figure 2, B and F), we quantified g-ratios across binned axon diameter groups (Figure 2, D and H). These groups include axons with diameters <0.5 μm (approx. bottom 10%), 0.5–1 μm (approx. bottom 10–50%), 1–2 μm (approx. top 10–50%), and ≥2 μm (approx. top 10%). At this early stage, the g-ratio did not differ significantly between the genotypes across any size groups, showing no evidence of significant myelin loss (Figure 2D). Importantly, the onset of motor impairments at 30 weeks, despite the absence of demyelination, suggests that myelin reduction is unlikely to be the primary driver of oligodendroglia-induced motor dysfunction in the early stages of the disease. Instead, these results suggest that PC axon shrinkage occurs non-cell-autonomously due to mutant ataxin-1 expression in oligodendroglia from the early stages of disease progression, independent of myelin loss, and is likely a key factor in the onset of early motor deficits.

At 80 weeks, the higher frequency of small-caliber axons persisted in OL-SCA1-cKI mice (Figure 2G). Furthermore, the g-ratio of the smallest axons (<0.5 μm) was significantly greater in OL-SCA1-cKI, with a similar trend observed in the 0.5–1 μm group, suggesting a preferential demyelination of thin axons (Figure 2H). Western blot analysis of bulk cerebellar tissue further suggested a progressive trending decline in myelin protein levels (Supplemental Figure 3). This later-stage demyelination mirrors previous findings in constitutive SCA1-KI mice (33), indicating that myelin deficits in SCA1 result from the cell-autonomous expression of mutant ataxin-1 in oligodendroglia. Together, these results establish that both myelin dysregulation and PC axon shrinkage are central pathological features in SCA1, driven by oligodendroglial mutant ataxin-1 expression. These findings highlight an underappreciated, direct role of oligodendroglia in cerebellar dysfunction, providing insight into SCA1 pathology.

### PC torpedoes and myelin debris in the granular layer are increased in OL-SCA1-cKI mice.

**Figure 3:**
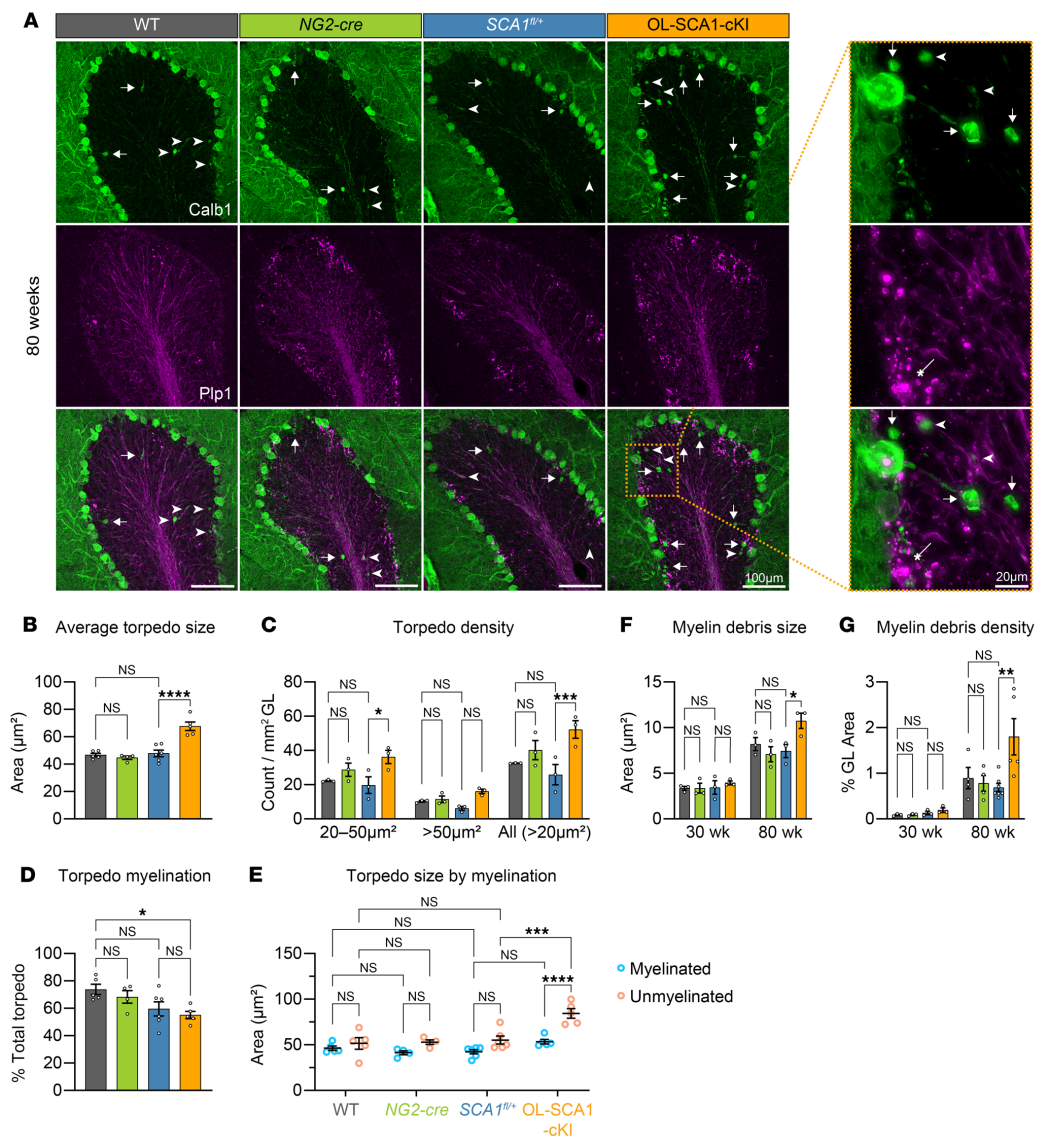
PC torpedoes and myelin debris in the granular layer are increased in OL-SCA1-cKI mice. (**A**) IHC of cerebellar sections from OL-SCA1-cKI and control mice at 80 weeks. Arrowheads indicate myelinated torpedoes, arrows indicate unmyelinated torpedoes, and asterisk-headed arrows indicate myelin debris. Scale bar: 100 μm (main) and 20 μm (enlarged). (**B** and **C**) Quantifications of PC torpedoes (>20 μm^2^) in size (**B**) and density (**C**) in the granular layer (GL). Data represent mean ± SEM, with *n* = 3–6 mice per genotype. One-way ANOVA (**B**) or 2-way ANOVA with Tukey’s test (**C**): *P < 0.05, ***P < 0.001, ****P < 0.0001. (**D** and **E**) Quantifications of PC torpedoes (>20 μm^2^) based on myelination status (**D**) and size differences between myelinated and unmyelinated (**E**) in OL-SCA1-cKI and control mice at 80 weeks. Data represent mean ± SEM, with *n* = 4–6 mice per genotype. Two-way ANOVA with Tukey’s test (**E**): *P < 0.05, ***P < 0.001, ****P < 0.0001. (**F** and **G**) Quantifications of Plp1^+^ myelin debris in size (**F**) and density (**G**) in the granular layer at 30 and 80 weeks. Data represent mean ± SEM, with *n* = 3–4 mice per genotype. Two-way ANOVA with Tukey’s test: *P < 0.05, **P < 0.01.

To further investigate PC damage in OL-SCA1-cKI mice, in addition to examining axon shrinkage, we assessed whether ataxin-1-induced oligodendroglial defects promote the formation of PC axonal torpedoes (also known as axon swelling or axonal spheroids), a hallmark pathology observed in SCA1 (33, 45). While axonal swelling is increased with normal aging (46–48), it is further exacerbated in various neurological diseases, including AD, MS, Parkinson’s disease, and essential tremor (48–51). In MS, demyelinated regions are strongly associated with increased axon swelling, suggesting a link between myelin health and axon swelling (51). While no Calb1^+^ PC axonal torpedoes were observed in any genotype at 30 weeks (Supplemental Figure 4), they became evident in the granular layer at 80 weeks across all genotypes (Figure 3A). Quantification of torpedoes (>20 μm^2^) revealed a significant increase in both size and density in OL-SCA1-cKI mice compared with controls at 80 weeks (Figure 3, B and C). Costaining with the myelin protein proteolipid protein 1 (Plp1) showed that a larger fraction of these torpedoes were unmyelinated in OL-SCA1-cKI mice (Figure 3D). Moreover, unmyelinated torpedoes were significantly larger than myelinated torpedoes in OL-SCA1-cKI mice, whereas no such difference was observed in controls (Figure 3E). Notably, we also observed a significant accumulation of myelin debris in the granular layer near the PC layer in OL-SCA1-cKI mice at 80 weeks but not at 30 weeks (Figure 3, F and G, and Supplemental Figure 4). Such myelin debris accumulation, previously reported in MS (52) and ALS (53), may destabilize axons at the nodes of Ranvier and exacerbate axonal damage (54). The progression from early distal axon shrinkage in the white matter at 30 weeks (Figure 2D) to late-stage proximal axon swelling in the granular layer at 80 weeks (Figure 3) aligns with the current model of spheroid formation (55, 56), supporting the idea that mutant ataxin-1–induced oligodendroglial dysfunction drives progressive axonal damage in SCA1.

Next, to evaluate the contribution of neurons to the severity of axonal pathology, we performed this experiment in *SCA1^fl/+^* mice crossed with *Nestin-cre* (*SCA1^fl/+^* *Nestin-cre*), in which recombination occurs throughout the CNS, including PCs and oligodendroglia (57, 58). These mice exhibited significant PC loss (Supplemental Figure 5A), characteristic intranuclear ataxin-1 inclusions (Supplemental Figure 5B), and reduced Calb1 expression in PC axons at 80 weeks (Supplemental Figure 5C). Notably, intranuclear ataxin-1 aggregates were detected only in PCs and not in the white matter of *SCA1^fl/+^* *Nestin-cre* mice (Supplemental Figure 5B). Next, we examined PC axonal phenotypes in these mice. Despite the technical limitation of reduced Calb1 expression in PC axons, we observed an increased number of Plp1^+^ myelinated torpedoes and myelin debris in both OL-SCA1-cKI and *SCA1^fl/+^* *Nestin-cre* mice (Supplemental Figure 5, C–F). Interestingly, axonal and myelin damage in OL-SCA1-cKI was comparable to that in *SCA1^fl/+^* *Nestin-cre* mice, whereas PC morphology, density, and Calb1 protein levels remained unchanged at 80 weeks in OL-SCA1-cKI mice (Figure 3A and Supplemental Figure 5, A and G–I). These results suggest that polyQ-expanded mutant ataxin-1 expression in oligodendroglia is sufficient to drive axonal damage but insufficient to induce PC soma degeneration.

We also examined astrocyte and microglial activation (gliosis) in OL-SCA1-cKI mice and found no evidence of gliosis in the white matter or granular layer at 80 weeks, despite the observed axonal and myelin damage (Supplemental Figure 6). Quantification of glial fibrillary acidic protein–positive (GFAP^+^) (astrocyte marker) and Iba1^+^ (microglial marker) signal intensities revealed no significant differences between genotypes (Supplemental Figure 6, B and C). These results suggest that the axonal damage observed in OL-SCA1-cKI mice is a direct consequence of oligodendroglial dysfunction, independent of secondary glial activation. In summary, mutant ataxin-1 expression in oligodendroglia drives progressive axonal pathology in SCA1, characterized by increased PC torpedoes and myelin debris, without inducing gliosis.

### The hippocampus exhibits accumulated myelin debris but not axonal swelling.

Given that oligodendroglia are ubiquitous throughout the CNS and SCA1 pathology affects multiple brain regions (45), we next investigated whether the myelin and axonal defects observed in the cerebellum also occur in extracerebellar areas. We observed a similar accumulation of myelin debris in the hippocampus of both OL-SCA1-cKI and *SCA1^fl/+^* *Nestin-cre* mice at 80 weeks compared with controls, whereas the cortex and brainstem showed minimal debris accumulation (Supplemental Figure 7, A–D). We then evaluated axon swelling in the hippocampus. Although Plp1^+^DAPI^–^ myelinated axonal swellings were detected, their density did not differ significantly across genotypes (Supplemental Figure 7, E and F). These results indicate that while mutant ataxin-1 expression in oligodendroglia promotes myelin debris accumulation in the hippocampus, PC axons are selectively vulnerable to oligodendroglial dysfunction.

### Non-cell-autonomous PC response to oligodendroglial dysfunction in SCA1.

**Figure 4:**
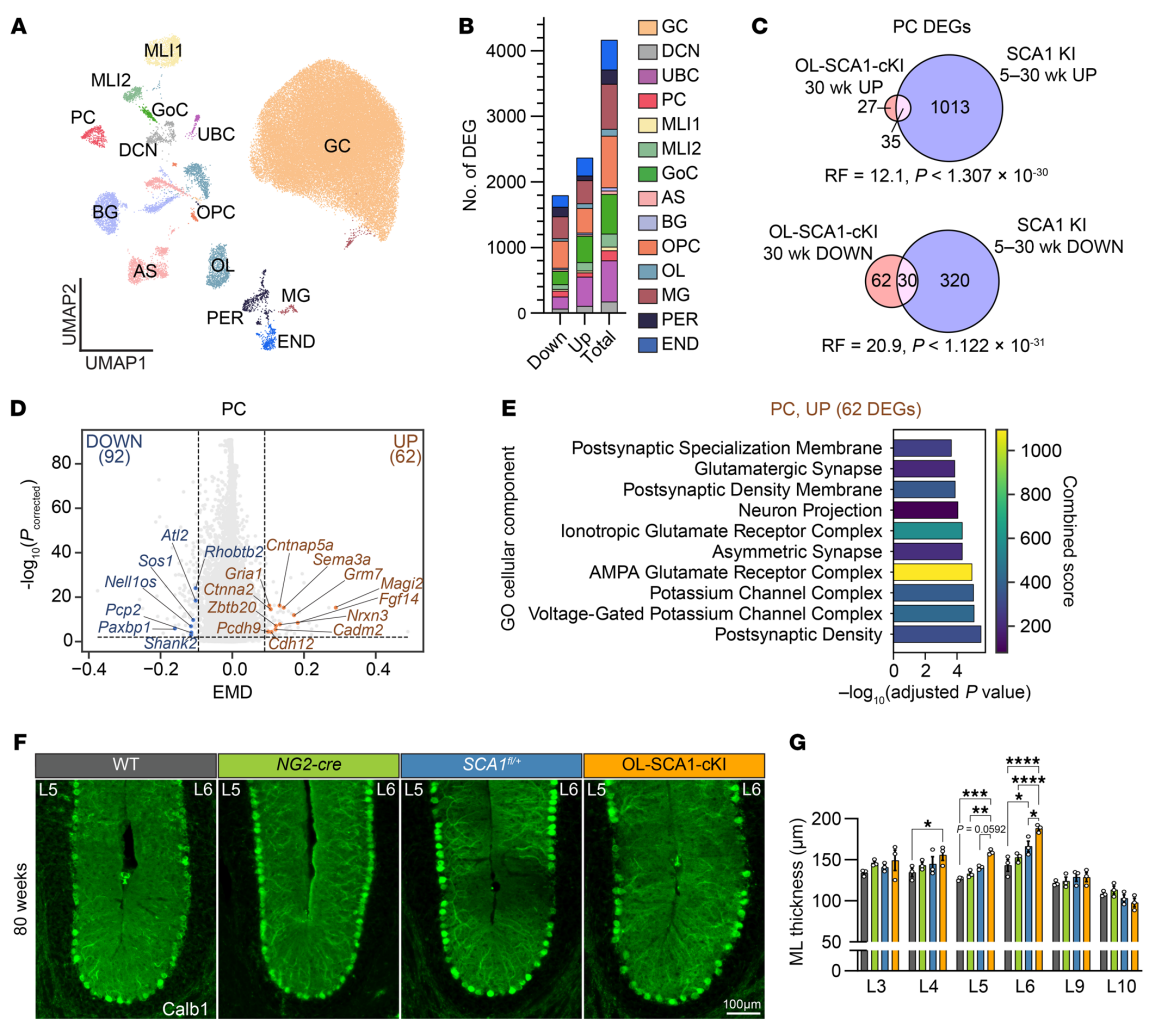
Non-cell-autonomous molecular and cellular PC response to OL-induced dysfunction in SCA1. (**A**) Uniform manifold approximation and projection (UMAP) plot of annotated cerebellar cell types from OL-SCA1-cKI and SCA1^fl/+^ littermate control mice at 30 weeks (*n* = 4 mice per genotype. SCA1^fl/+^, *n* = 28,908; OL-SCA1-cKI, *n* = 35,396 nuclei). (**B**) Bar graphs depicting the number of downregulated (Down), upregulated (Up), and total DEGs across cerebellar cell types. DEGs were considered significant with *P*_corrected_ < 0.05 and imputed |EMD| > 0.1. GC, granule cell; DCN, deep cerebellar nuclei neuron; UBC, unipolar brush cell; PC, Purkinje cell; MLI1, molecular layer interneuron 1; MLI2, molecular layer interneuron 2; GoC, Golgi cell; AS, astrocyte; BG, Bergmann glia; OPC, oligodendrocyte progenitor cell; OL, oligodendrocyte; MG, microglia; PER, pericyte; END, endothelial cell; EMD, Earth Mover’s distance. (**C**) Venn diagrams illustrating the overlap of PC DEGs between OL-SCA1-cKI and constitutive SCA1-KI datasets (33). Representation factors (RF; observed/expected number of overlapping genes) and *P* values were calculated using a hypergeometric test. (**D**) Volcano plot of PC DEGs from OL-SCA1-cKI mice, highlighting genes overlapping with those in constitutive SCA1-KI. DOWN, downregulated genes; UP, upregulated genes. (**E**) GO analysis of 62 upregulated PC DEGs, highlighting cellular components associated with postsynaptic function. (**F**) Representative IHC images of cerebellar lobules (L5 and L6) in 80-week-old OL-SCA1-cKI and control mice. (**G**) Quantification of molecular layer (ML) thickness in anterior (L3 and L4), medial (L5 and L6), and posterior (L9 and L10) cerebellar regions. Data represent mean ± SEM with *n* = 3 mice per genotype. Two-way ANOVA with Tukey’s test: *P < 0.05, **P < 0.01, ***P < 0.001, ****P < 0.0001.

To dissect the early molecular mechanisms by which mutant ataxin-1 disrupts OPC and OL, as well as how these alterations propagate across cerebellar cell types, particularly PC, we performed snRNA-seq on the cerebella of OL-SCA1-cKI mice and their *SCA1^fl/+^* littermate controls at 30 weeks. After applying stringent quality control pre-processing, we identified distinct clusters of nuclei corresponding to major neuronal and glial populations based on established marker gene expression (33) (Figure 4A and Supplemental Figure 8, A–D). Notably, the proportions of OPCs, OLs, PCs, and other cerebellar cell types remained unchanged (Supplemental Figure 8E), indicating that oligodendroglial expression of mutant ataxin-1 in OL-SCA1-cKI mice is insufficient to trigger PC death. Furthermore, consistent with the snRNA-seq results, in vivo assessment of proliferation using 5-ethynyl 2′-deoxyuridine (EdU) labeling revealed no differences in the proliferation rate of Pdgfra^+^ OPCs across the CNS (Supplemental Figure 9), suggesting that OPC proliferative capacity is unaffected.

To pinpoint cell type–specific gene expression changes, we identified differentially expressed genes (DEGs) (Figure 4B and Supplemental Table 1). Notably, the non-cell-autonomously induced DEGs in OL-SCA1-cKI PCs exhibited significant overlap with those in constitutive SCA1-KI mice (33) (Figure 4, C and D), highlighting a shared pathological molecular signature. Gene Ontology (GO) analysis revealed an upregulation of pathways related to postsynaptic organization (Figure 4E), while no significant enrichment was observed in downregulated genes, suggesting aberrant synaptic remodeling as an early transcriptomic response to oligodendroglial dysfunction. At the structural level, this was mirrored by increased PC dendritic length, as inferred from molecular layer thickness in the medial cerebellar lobules at 80 weeks (Figure 4, F and G) but not at 30 weeks (Supplemental Figure 8F). These findings are consistent with the previous report of increased parallel fiber-PC dendritic synapses in SCA1-KI (33) and the established role of OL in modulating dendritic spine turnover (59) and PC dendritic arborization (60). Interestingly, this compensatory PC dendritic arborization was not observed in the posterior lobules, which are preferentially and more severely affected in SCA1 (61–64). Collectively, our results highlight a crucial, non-cell-autonomous role of OL dysfunction in driving PC gene dysregulation in SCA1. The observed dendritic compensation in PCs likely represents an adaptive response to axonal dysfunction, reinforcing the central role of OPCs and OLs in cerebellar pathology.

### Oligodendrocyte subtype imbalance and dysfunction drive SCA1 pathogenesis.

**Figure 5:**
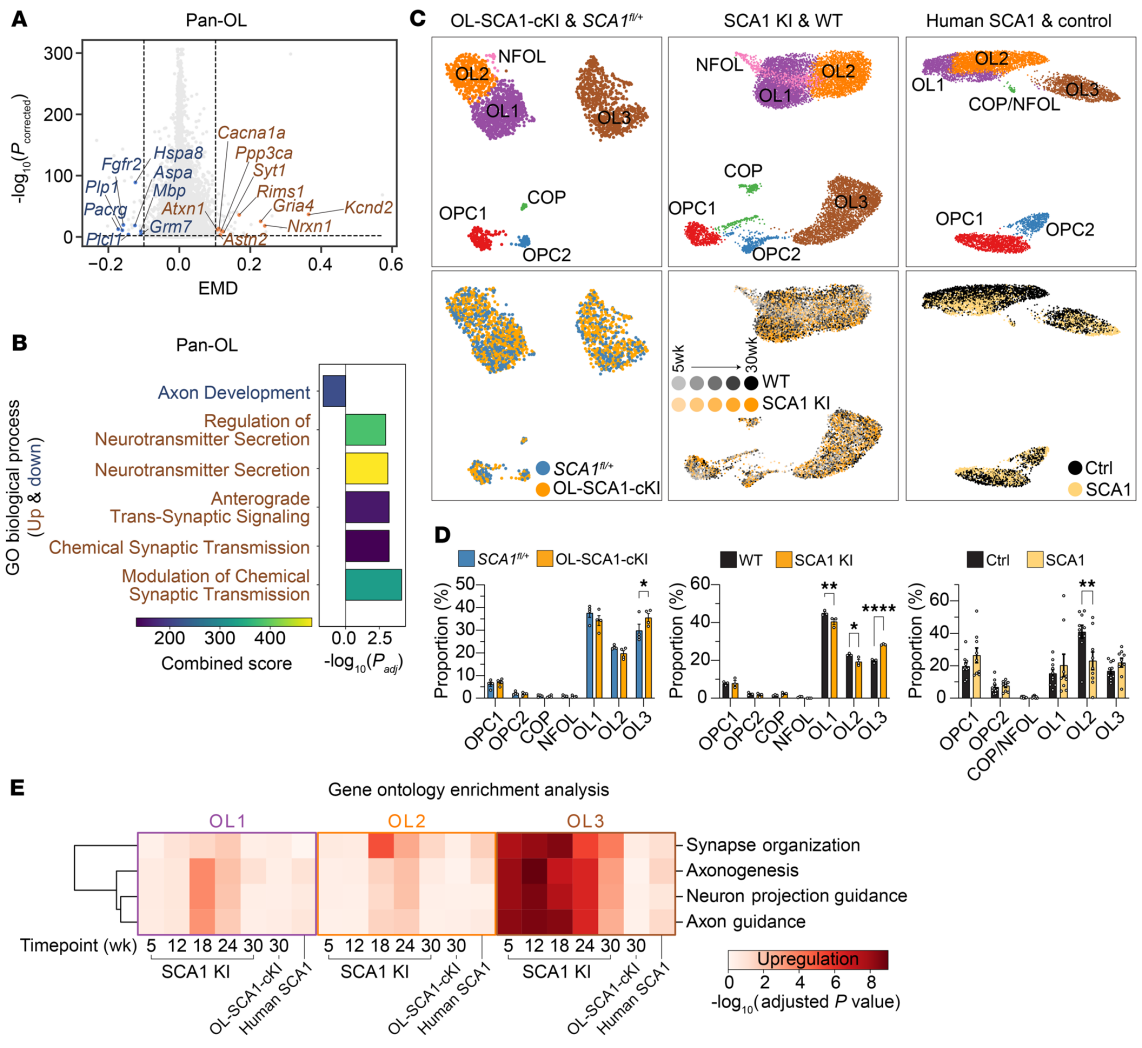
OL subtype imbalance and dysfunction drive SCA1 pathogenesis. (**A**) Volcano plot of pan-OL DEGs from OL-SCA1-cKI mice, highlighting genes overlapping with those in constitutive SCA1-KI. (**B**) GO analysis of pan-OL DEGs, highlighting downregulation of axon development pathway and upregulation of axo-myelinic synapse-related pathways. (**C**) UMAP plots illustrating oligodendroglial populations by identified subtype (top) and by genotype and time point (bottom) across datasets: (left, OL-SCA1-cKI; middle, SCA1-KI; right, SCA1 human samples). (**D**) Bar graphs quantifying oligodendroglial subtype proportions in OL-SCA1-cKI (left; *n* = 4 mice per genotype), SCA1-KI at 24 weeks (middle; *n* = 3 mice per genotype), and SCA1 human (*n* = 9) and healthy control (*n* = 10) samples (right). Data are presented as mean ± SEM. Two-way ANOVA with Tukey’s test: *P < 0.05, **P < 0.01, ****P < 0.0001. (**E**) GO analysis of upregulated DEGs in OL1-3 across SCA1-KI, OL-SCA1-cKI, and human SCA1 datasets. Upregulated genes in OL3 are associated with axonogenesis and axonal support, a compensatory response that declines over disease progression.

OLs showed downregulation of key genes involved in myelin formation and axon maintenance, including *Plp1*, *Mbp*, *Aspa*, *Grm7* (65), and *Fgfr2* (66) (Figure 5A and Supplemental Table 1). Given that myelin proteins have long half-lives of approximately 6 months and slow turnover rates (67, 68), this transcriptional downregulation, despite the observed intact myelination at the ultrastructural level (Figure 2, A–D), suggests that a reduction in myelin production is initiated around this stage, ultimately contributing to demyelination at later time points (Figure 2, E and G). Importantly, GO analysis also highlighted downregulation of axon development (Figure 5B), consistent with the observed axon shrinkage (Figure 2, D and H). These results implicate early transcriptional dysregulation of myelin- and axon-supporting genes in driving both cell-autonomous demyelination and non-cell-autonomous PC axonal pathology in SCA1.

Intriguingly, GO analysis further revealed an upregulation of synapse-related pathways in OLs, suggesting potential compensatory alterations in neuron-OL communication at axo-myelinic synapses (69, 70) (Figure 5B). Recent studies have demonstrated that oligodendroglia exhibit distinct transcriptomic subtypes (71, 72), each with unique responses to disease (23, 73, 74). We hypothesized that OL subpopulations in SCA1 may exhibit divergent responses, including contributions to neurotoxic and neuroprotective effects. To investigate this, we subclustered OPCs and OLs to identify transcriptionally distinct subtypes within the OL lineage. In addition to the OL-SCA1-cKI dataset, we reanalyzed previously generated human SCA1 and constitutive SCA1-KI mouse snRNA-seq datasets (33) using an updated analysis pipeline. Subclustering across all datasets identified 2 OPC subtypes (*Caln1[CALN1]*^lo^ OPC1 and *Caln1[CALN1]*^hi^ OPC2), committed OPC (COP), newly formed OL (NFOL), and 3 mature OL subtypes (OL1, OL2, OL3) (Figure 5C and Supplemental Figure 10, A and B). Notably, *Ptgds*(*PTGDS*)^hi^ OL1 and *Klk6*(*KLK6*)^hi^ OL2 corresponded to mature OL subtypes found throughout the CNS (MOL5/6 and MOL2, respectively) (23, 72). In contrast, *Caln1*(*CALN1*)^hi^ OPC2 and *Fgf14*(*FGF14*)^hi^ *Rbfox1*(*RBFOX1*)^hi^ OL3 exhibited significant cerebellum-specific enrichment compared with the cortex and striatum (Supplemental Figure 11, A and C). Further analysis of an independent mouse cerebellum snRNA-seq dataset (75) confirmed an even distribution of these OL subpopulations across cerebellar subregions (Supplemental Figure 11, B–D). GO analysis of the top 100 genes per subtype, derived from WT mice or control human samples, revealed distinct functional specializations: OL2 was highly enriched in myelin formation pathways, while OL3 was associated with synaptic signaling, implicating OL3’s role in axo-myelinic synapses (Supplemental Figure 10C). These findings underscore the transcriptomic heterogeneity of cerebellar oligodendroglia, providing a crucial foundation for downstream analyses.

To determine whether SCA1 disrupts OL subtype composition, we examined the relative abundance of OL subtypes in both human and mouse SCA1 samples. The myelin gene–enriched OL2 subtype was significantly reduced in human SCA1 and SCA1-KI datasets, whereas it showed a nonsignificant decrease in the OL-SCA1-cKI dataset (Figure 5D), implicating its loss as a key driver of SCA1-associated demyelination (33). In contrast, the cerebellum-specific OL3 was significantly increased in both OL-SCA1-cKI and SCA1-KI datasets, suggesting that this expansion represents an early compensatory response to disease progression. Collectively, these findings indicate that an imbalance in OL subtypes underlies SCA1-related OL dysfunction in the cerebellum.

To uncover the functional dysregulation of oligodendroglial subtypes and their specific contributions to SCA1, we identified DEGs for each subtype (Supplemental Table 2). In mouse OL subtypes, *Atxn1* expression varied among subtypes, with minimal DEG overlaps across mature OLs. In contrast, human OL subtypes exhibited more uniform *ATXN1* expressions and greater DEG overlap (Supplemental Figure 12). GO pathway analysis further highlighted distinct dysregulated biological processes across SCA1 OL subtypes (Supplemental Figure 13 and Supplemental Table 3). OL1 and OL2 showed a pronounced reduction in genes critical for small GTPase-mediated intracellular signal transduction (e.g., *DOCK3*, *PREX1*, *PREX2*, *FGD4*, *RHOU*, etc.) in human SCA1. This signaling pathway is essential for oligodendroglia to respond to neuronal activity and provide proper myelination (76). Given that impaired neuronal activity detection can also compromise metabolic support to neurons (77), our findings suggest that OL1 and OL2 become less responsive to neuronal signals, leading to widespread deficits in myelination and axonal support in SCA1. In contrast, OL3 showed a striking upregulation of genes involved in axonogenesis, axon guidance, synaptic organization, and neuron projection guidance across all datasets (Figure 5E and Supplemental Figure 13), suggesting a compensatory neuroprotective response, via enhanced axonal support or increased OL3 abundance (Figure 5D). Notably, longitudinal constitutive SCA1-KI mouse data revealed that this upregulation is most pronounced at early disease stages but declines over time (Figure 5E), indicating a progressive loss of compensatory capacity of cerebellum-specific OL3, which may contribute to disease exacerbation. Together, these findings reveal a dual mechanism underlying OL dysfunction in SCA1: impaired OL1/OL2-mediated myelination and axonal support, coupled with OL3’s diminishing neuroprotective response over disease progression.

### TCF7L2 and HTT as potential early drivers of dysregulated OL-neuron communication in SCA1.

**Figure 6:**
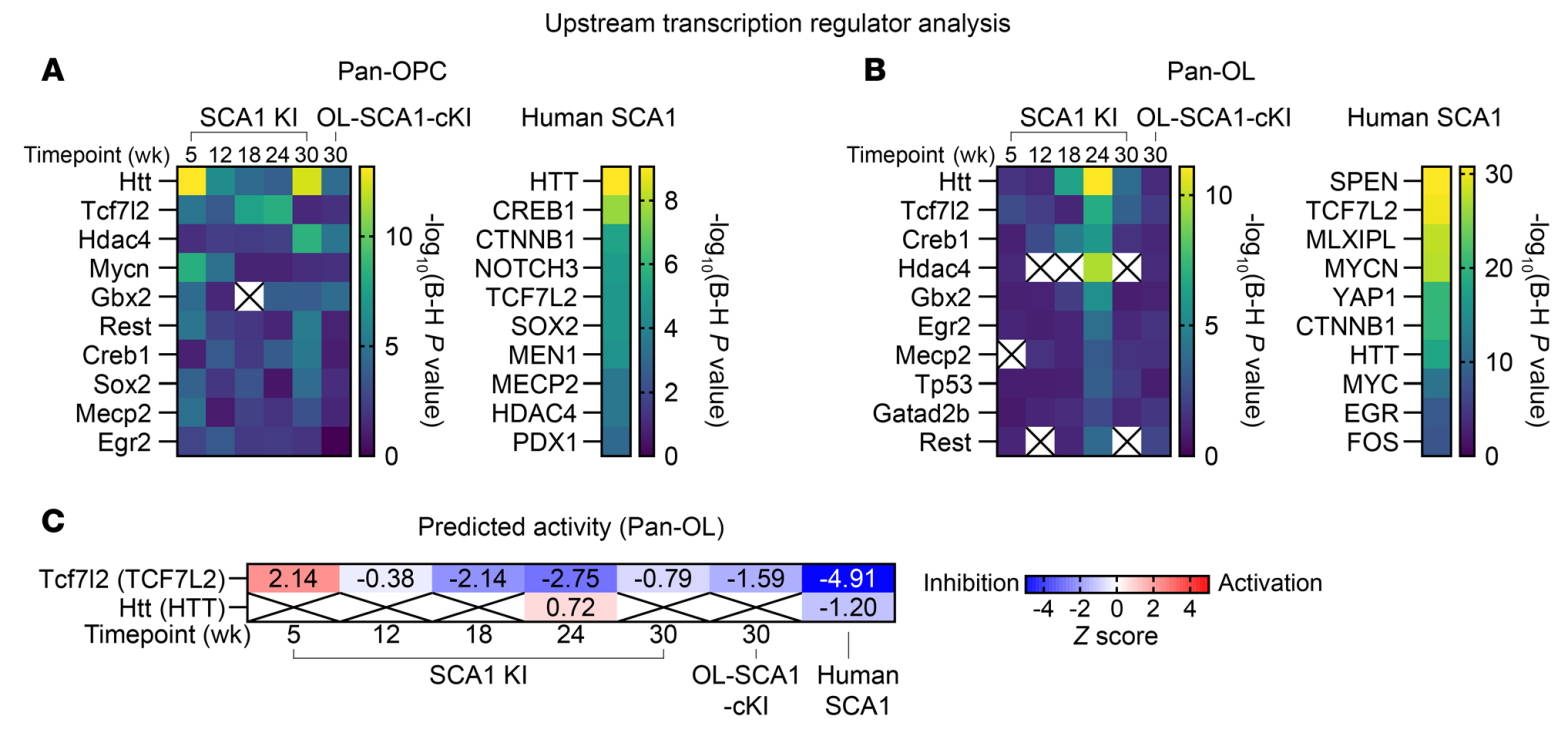
TCF7L2 and HTT as potential early drivers of dysregulated OL-neuron communication in SCA1. (**A** and **B**) Upstream transcription regulator analysis (QIAGEN IPA) of DEGs from pan-OPC (**A**) and pan-OL (**B**) identifies TCF7L2 and HTT as top key regulators in both human and mouse SCA1 datasets. (**C**) Activation *Z*-scores of TCF7L2 and HTT, determined by IPA using both up- and downregulated DEGs from pan-OL. Positive *Z*-scores indicate predicted activation, while negative *Z*-scores indicate predicted inhibition.

To identify upstream transcriptional regulators driving gene expression changes and OL subtype imbalances in SCA1, we performed QIAGEN Ingenuity Pathway Analysis (IPA) using both upregulated and downregulated DEGs from pan-OPC and pan-OL populations (Supplemental Tables 4 and 5). This analysis highlighted TCF7L2 and HTT as key regulators across all datasets (Figure 6, A and B). TCF7L2 (also known as TCF4) is a critical mediator of the Wnt/β-catenin pathway and plays a central role in OL differentiation and (re)myelination (78). Our previous work demonstrated Wnt/β-catenin signaling dysregulation in SCA1 Bergmann glia and a physical interaction between polyQ-expanded ataxin-1 and TCF7L2 (61). Interestingly, while TCF7L2 transcriptional activity is predicted to be suppressed in OLs (Figure 6C), its gene expression is upregulated in COP/NFOL and OL1-3 populations (Supplemental Figure 14), suggesting that polyQ-expanded ataxin-1 may interfere with TCF7L2 function, triggering a compensatory increase in *TCF7L2* mRNA levels. Similarly, HTT, well known for its role in HD, has been implicated in OL dysfunction, demyelination, and impaired differentiation (79). Although further studies are needed to define the precise molecular interactions, the identification of TCF7L2 and HTT as potential molecular intermediaries highlights these pathways as promising targets for restoring OL function in SCA1.

## Discussion

While alterations in oligodendroglia have been reported in many neurodegenerative diseases (6–13, 23–28, 73, 74, 79, 80), including SCA1 (4, 5, 33), it remains largely unclear whether these changes actively drive disease progression or arise as secondary consequences. In this study, by employing an oligodendroglia-specific SCA1 conditional knockin mouse model (OL-SCA1-cKI), we provide compelling evidence that oligodendroglia play a direct pathogenic role in SCA1. Our findings establish oligodendroglia as key contributors to SCA1 pathology by redefining their function beyond passive bystanders. We demonstrate that mutant ataxin-1 expression in oligodendroglia is sufficient to induce motor dysfunction and significant axonal and myelin pathology, as evidenced by biochemical, histological, and ultrastructural assays. Single-nucleus transcriptomic analyses further reveal distinct, cell type–specific disruptions in cerebellar cell populations, including PCs and OLs, providing crucial insights into the molecular underpinnings of SCA1 pathology.

Our long-term analysis of the OL-SCA1-cKI model offered a unique opportunity to examine the prolonged effects of polyQ ataxin-1 on key pathological hallmarks of SCA1, including PC torpedo formation and myelin debris accumulation at 80 weeks of age. These findings are particularly striking, as commonly used SCA1-KI models exhibit premature mortality by 45 weeks (30), precluding observation of late-stage pathology. Moreover, our TEM data reveal that while control *SCA1^fl/+^* mice exhibit age-related thickening of myelin, OL-SCA1-cKI mice fail to sustain myelination, ultimately leading to demyelination of thin axons. These results reinforce the notion that intrinsic oligodendroglial pathology actively contributes to myelin loss in SCA1 (33).

Notably, our data suggest that while demyelination compromises axonal insulation and support, early PC axonal shrinkage may play a primary role in driving motor deficits. Beyond myelination, OLs provide essential metabolic support to axons through direct contact (14, 81). Although overt structural myelin deficits are absent in OL-SCA1-cKI mice, dysregulation of genes involved in axon development and axo-myelinic synapses indicates impaired signaling and metabolic support, which may lead to deficits in axonal transport, action potential propagation, and synaptic function (82). Future metabolomic and electrophysiological studies will be essential to validate these predicted functional impairments.

In addition to identifying cerebellum-specific oligodendroglia subtypes and their dysfunction, a particularly compelling discovery is the downregulation of *PLP1*(*Plp1*) across both human and mouse models of SCA1. Our findings parallel those in Plp1-deficient mice, which exhibit axonal swelling and shrinkage despite preserved myelin coverage, an effect not observed in mice lacking Mbp (67, 83). This suggests that mutant ataxin-1 disrupts axonal integrity through Plp1-dependent mechanisms, warranting further investigation into whether restoring Plp1 expression can ameliorate SCA1-related axonal and motor deficits. However, the therapeutic window must be carefully defined, as excessive Plp1 overexpression could exacerbate myelin debris accumulation. Furthermore, the notion that dysfunctional myelin may be more detrimental to axons than complete myelin loss (84, 85) introduces a dimension to our understanding of how OL dysfunction drives axonal degeneration in SCA1.

Our study also identifies TCF7L2 and HTT as potential upstream regulators of the oligodendroglial dysfunction in SCA1, suggesting common molecular mechanisms underlying OL pathology across polyQ diseases. Notably, the YAC-Q84/Q84 mouse model of SCA3, another polyQ disorder, similarly exhibits delayed OL differentiation and demyelination, with RNA-seq analyses pinpointing TCF7L2 and HTT as key regulatory factors (86). Given the established role of Wnt/β-catenin signaling in SCA1 (61) and HD (87) and its therapeutic potential in disease models (88), it remains to be determined whether polyQ-expanded mutant ataxin-1 interacts with HTT or its complexes (89) in OLs to drive similar pathological cascades. These findings underscore the broader relevance of Wnt/β-catenin signaling in polyQ disorders and warrant comparative analyses across related neurodegenerative diseases.

Despite the strength of our findings, certain limitations must be considered. First, the unchanged abundance of OPCs and mature OLs in OL-SCA1-cKI snRNA-seq data suggests that OPC-to-OL differentiation remains largely intact in this model, in contrast with the partial differentiation block observed in constitutive SCA1-KI mice (33). This is consistent with the preservation of the OL2 subpopulation in OL-SCA1-cKI mice, whereas OL2 is significantly reduced in both constitutive SCA1-KI mice and human patients. This discrepancy may reflect the lower expression levels of mutant ataxin-1 in the conditional *SCA1^fl/+^* allele compared with the SCA1-KI mice (34), suggesting that higher mutant ataxin-1 expression in oligodendroglia might be required to induce more severe differentiation impairments and earlier-onset pathology. Alternatively, differentiation defects in SCA1-KI mice may result from mutant ataxin-1 expression in non-oligodendroglial cells, potentially influenced by altered neuronal activity from PCs. Second, although our study primarily focuses on cerebellar pathology and motor deficits, we also observed significant myelin debris deposition in the hippocampus (Supplemental Figure 7). The precise role of oligodendroglia in extracerebellar regions and their potential contributions to broader clinical manifestations, such as cognitive impairments (90), remain largely unexplored. Addressing this gap could provide critical insights into the systemic impact of oligodendroglial dysfunction in SCA1.

In conclusion, our study establishes oligodendroglial dysfunction as a central driver of SCA1 pathology, profoundly impacting myelin integrity, axonal health, and motor function. By mapping the gene expression changes and subtype-specific vulnerabilities underlying SCA1, we provide a foundation for future investigations into the molecular mechanisms of disease progression. Importantly, these insights pave the way for the development of targeted therapeutic strategies aimed at mitigating oligodendroglial pathology and preserving neuronal function in SCA1 and potentially in other polyQ diseases.

## Methods

### Sex as a biological variable

Our study examined male and female animals, and potential sex effects were minimized by either balancing group composition (50% male, 50% female) or by calculating weighted means and SEM to equally represent both sexes (see Statistics).

### Animal husbandry

Mice were housed under a 12-hour light/dark cycle, provided standard laboratory chow, and given ad libitum access to water. Female *Cspg4* *(NG2)-cre* mice (Jackson Laboratories, 08533) (35, 36) and *Nestin-cre* mice (Jackson Laboratories, 003771) (57, 58), were purchased and backcrossed for at least 3 generations to establish a C57N3 background. Male SCA1 conditional KI (*SCA1^fl/+^*) mice on pure C57BL/6J background (34) were crossed with female *NG2-cre/^+^* or *Nestin-cre/^+^* mice on a C57 background (≥N3) to generate WT, *NG2-cre*, *Nestin-cre*, *SCA1^fl/+^*, OL-SCA1-cKI (*SCA1^fl/+^ NG2-cre*), and *SCA1^fl/+^ Nestin-cre*. These mouse lines were maintained on a C57 background (≥N4). Ai9 Cre reporter mice (Jackson Laboratories, 007909) were also used in this study. For genotyping, tail biopsies were collected for DNA extraction before postnatal day 17. Body weight was measured every 2 weeks starting at 5 weeks of age. For survival assessment, lifespan was defined as the time to natural death or euthanasia upon reaching humane endpoints, in accordance with guidelines established by the Yale University Institutional Animal Care and Use Committee (IACUC).

### Mouse behavioral analysis

Mice were habituated to the testing room for 1 hour before the experiments. Gait impairments and motor coordination were evaluated using the accelerating rotarod, dowel rod, and wire hang tests, as previously described (39–41). The rotarod apparatus was set to an initial speed of 4 rpm, gradually increasing to 40 rpm over 5 minutes, followed by an additional 5 minutes at 40 rpm. The latency to fall was recorded for each trial. Three full rotations without a step were considered a fall. Mice underwent 4 trials per day, with a 30-minute interval between trials, for 4 consecutive days. For the wire hang or dowel rod tests, mice were placed at the center of a suspended wire or a 9 mm dowel rod connected to platforms at both ends. The time taken to reach and touch the adjacent platform for the first time, the time to first fall, and the total number of side touches and falls within a 120-second observation period were recorded.

### Electron microscopy

Mice were anesthetized with isoflurane and transcardially perfused with 1× phosphate-buffered saline (PBS) for 1 minute, followed by 4% paraformaldehyde (PFA) in PBS for 4 minutes and a final rinse with 1× PBS for an additional minute. Cerebella were sagittally cut into hemispheres and fixed overnight at 4°C in 2.5% glutaraldehyde and 2% PFA in 0.1M sodium cacodylate, pH 7.4. Each fixed cerebellum hemisphere was coronally sectioned into 150 μm slices in cold PBS using a vibratome (Leica VT1000) and postfixed in 1% OsO_4_ and 0.8% potassium ferricyanide in 0.1M sodium cacodylate, pH 7.4, for 1 hour on a shaker at room temperature (RT), followed by 3 washes in 0.1M sodium cacodylate, pH 7.4, for 5 minutes each on a shaker at RT. Specimens were stained en bloc with 2% aqueous uranyl acetate for 30 minutes, dehydrated through a graded ethanol series to 100%, substituted with propylene oxide, and embedded in Embed resin. Sample blocks were polymerized overnight at 60°C. Then 60 nm sections of the micro-dissected white matter were prepared using a UC7 ultramicrotome (Leica) and poststained with 2% uranyl acetate and lead citrate. Sections were examined using an FEI Tecnai transmission electron microscope at 90 kV accelerating voltage, and images were captured with an Olympus Morada CCD camera and iTEM imaging software at the Yale University CCMI Electron Microscopy Facility. The g-ratio was calculated by dividing the inner axonal diameter by the total outer diameter of the myelinated fiber.

### Protein extraction and Western blot analysis

Cerebellar tissue was homogenized using either Triple Lysis Buffer (0.5% NP-40, 0.5% Triton X-100, 0.1% SDS, 20 mM Tris-HCl pH 8.0, 180 mM NaCl, 1 mM EDTA) or RIPA Buffer (1% NP-40, 0.1% SDS, 50 mM Tris-HCl pH 8.0, 150 mM NaCl, 0.5% sodium deoxycholate), both with Roche Complete Protease Inhibitor Cocktail and PhosStop Protease Inhibitors, on ice using a Dounce homogenizer. Samples were then sonicated and rotated at 4°C for 10 minutes. The lysates were centrifuged at 15,871 × *g* for 10 minutes at 4°C, and the supernatants were collected. Protein concentrations were determined using a BCA assay (Thermo Fisher Scientific 23225). Equal protein amounts were denatured by boiling at 95°C for 10 minutes before electrophoresis on a gel (BioRad) at 120 V. Proteins were transferred onto nitrocellulose membranes (0.22 or 0.45 μm) at 100 V for 1 hour at 4°C. Membranes were washed 3 times with TBST (Tris-buffered saline, 0.1% Tween-20) for 10 minutes each, followed by blocking in 5% nonfat dry milk in TBST for 1 hour at RT, and membranes were incubated overnight at 4°C with primary antibodies diluted in 5% nonfat dry milk in TBST. The following day, membranes were washed 3 times with TBST for 10 minutes each and incubated for 2 hours at RT with HRP-conjugated secondary antibodies, including sheep anti-mouse IgG-HRP (MilliporeSigma, GENA931, 1:4,000) or donkey anti-rabbit IgG-HRP (MilliporeSigma, GENA934, 1:4,000). Membranes were then washed 3 times with TBST for 10 minutes each and developed using SuperSignal West Pico Plus Chemiluminescent Substrate (Pierce, 34580). Chemiluminescent signals were visualized using a KwikQuant Imager (Kindle Biosciences) and quantified using ImageStudioLite. The following primary antibodies were used: mouse anti-Vinculin (Millipore V9264, 1:10,000), mouse anti-Mog (Millipore MAB5680, 1:1,000), mouse anti–Calbindin-D-28K (Sigma C9848, 1:1,000), mouse anti-CNP (Abcam ab6319, 1:500), mouse anti-Mbp (Abcam ab40390, 1:1,000), mouse anti-Plp1 (Thermo Fisher Scientific MA1-80034, 1:300), rabbit anti–Ataxin-1 (11750, 1:1,000), rabbit anti-Mag (Invitrogen 34-6200, 1:1,000), and rabbit anti-gamma Tubulin (Sigma T6557, 1:10,000).

### IHC

Mice were anesthetized with isoflurane and transcardially perfused with 1× PBS for 1 minute, followed by 4% PFA in PBS for 4 minutes, and a rinse with 1× PBS for an additional minute. Whole brains were extracted and postfixed overnight in 4% PFA, then subsequently incubated in 20% and 30% sucrose solutions in PBS. The samples were then embedded in OCT compound (VWR, 4538), frozen, and sectioned into 30 μm slices using a cryostat (Leica). Free-floating sections were washed once in PBS for 5 minutes and twice with PBS-X (0.25% Triton-X in PBS) for 5 minutes each, followed by blocking in 5% normal goat serum (Jackson Laboratories, 005-000-121) at RT for 1 hour. Primary antibody incubation was performed at 4°C overnight with the following antibodies: mouse anti–Calbindin-D-28K (Sigma C9848, 1:1,000), rabbit anti-Plp1 (Abcam ab28486, 1:1,000), chicken anti-GFAP (Abcam ab4674, 1:1,000), rabbit anti-Iba1 (Wako 019-19741, 1:500), rabbit anti-Sox10 (Abcam ab227680, 1:100), rabbit anti-Nf1a (Sigma HPA006111, 1:500), rabbit anti-PDGFRA (Cell Signaling 3174, 1:1,000), mouse anti-APC[CC1] (Abcam ab16794, 1:250), rabbit anti-TCF4/TCF7L2 (Cell Signaling 2569, 1:500), mouse anti–Ataxin-1[11NQ N76/3] (Antibodies Incorporated 75-122, 1:500), goat anti–Calbindin-D-28K (Santa Cruz SC-7691, 1:500), and mouse anti–Neurofilament H (NF-H; SMI32) (BioLegend 801701, 1:500). After primary antibody incubation, sections were washed 3 times in PBS-X for 5 minutes each and incubated with secondary antibodies (Invitrogen Alexa Fluor, 1:500, A11001, A11011, A11012, A11057, A21206, A21237, and A21447) for 1.5 hours. After 2 washes in PBS-X and 1 wash in PBS for 5 minutes each, sections were mounted onto slides and coverslipped with VECTASHIELD mounting media containing DAPI (Vector Laboratories, H-1500). Fluorescent images were acquired using a Zeiss LSM880 confocal microscope or an Olympus VS200 slide scanner at 10×, 20×, or 40× original magnifications, maintaining consistent microscope settings across experiments. For each mouse, 3 to 6 brain sections were imaged and quantified.

### Fluorescent image quantification

For the Ai9 Cre-reporter analysis, CellProfiler (Broad Institute) was used to identify antibody-positive cells (Sox10, Calb1, Nf1a, Iba1, Pdgfra, Tcf7l2, or CC1) and RFP-positive cells. Colocalization percentages were determined using Manders’ overlap coefficient. PC numbers, torpedoes, and myelin debris were quantified using ImageJ (NIH). Image *z*-stacks were flattened to maximum-intensity *z*-projections, converted to 8-bit images, and thresholded using identical acquisition and processing parameters across all images. The granular layer was identified using DAPI-dense areas within the cerebellum. Gliosis was assessed by selecting cerebellar layers using DAPI staining. Fluorescence intensity of GFAP and Iba1 was quantified. Molecular layer thickness was measured by averaging PC dendrite lengths at 3 locations within the specified cerebellar lobule, from the apical tip of the PC soma to the end of the molecular layer. Images were thresholded to remove background intensity. For all quantifications, 3 to 6 images per mouse were analyzed.

### EdU cell proliferation assay

EdU (Thermo Fisher Scientific A10044) was dissolved in sterile PBS to a concentration of 5 mg/mL. Mice received intraperitoneal injections of 200 μL EdU solution daily for 3 consecutive days. Two hours following the final injection, mice were perfused, and brain sections were prepared as described above. EdU labeling was visualized using the Click-iT EdU Cell Proliferation Kit for Imaging (Alexa Fluor 488; Thermo Fisher Scientific C10337) according to the manufacturer’s instructions. The click chemistry detection was performed prior to the primary antibody incubation for standard IHC.

### Isolation of nuclei from frozen tissue

Nuclei isolation from frozen tissue followed a previously established protocol (91). Cerebellar hemispheres from 4 animals (2 males and 2 females) per genotype (*SCA1^fl/+^* and OL-SCA1-cKI) were collected at 30 weeks. Briefly, frozen tissues were gently homogenized using Dounce homogenizer in 2 mL of ice-cold Nuclei EZ Prep buffer (Sigma; NUC101-1KT) with a large clearance pestle A, followed by a small clearance pestle B, each for 25 strokes. The homogenized tissue was then incubated on ice for 5 minutes with an additional 2 mL of cold EZ Prep buffer. Following incubation, the homogenates were centrifuged at 500*g* for 5 minutes at 4°C. The nuclear pellets were resuspended in 4 mL of cold EZ Prep buffer, incubated on ice for 5 minutes, and centrifuged again at 500*g* for 5 minutes at 4°C. The nuclei were then washed in 4 mL of Nuclei Suspension Buffer (NSB), containing 1× PBS, 0.01% BSA, and 0.1% RNase inhibitor (Clontech/Takara; 2313B). The final washing step was followed by centrifugation at 500*g* for 5 minutes at 4°C. The purified nuclei were resuspended in 1 mL of NSB, filtered through a 40 μm cell strainer (Thermo Fisher Scientific; 22-363-547), and quantified using a Countess III FL Cell Counter (Thermo Fisher Scientific; AMQAF2000) with trypan blue. Single-nucleus suspensions were then diluted to approximately 1,000 nuclei/μL in NSB for library preparation.

### 10x Genomics snRNA-seq

Libraries were prepared from diluted single-nucleus suspensions using the 10x Genomics Chromium Single Cell 3′ Reagent Kits v.3.1 at the Yale Center for Genome Analysis (YCGA). Briefly, 10,000 cells per sample were mixed with RT Master Mix, loaded onto a Single Cell A Chip, and combined with approximately 750,000 barcoded gel beads to form nanoliter-scale Gel Beads-In-Emulsions (GEMs). Each gel bead contained primers with the following components: an Illumina R1 sequence, a 16-nucleotide barcode, a 12-nucleotide unique molecular identifier (UMI), and a 30-nucleotide poly-dT primer sequence. Upon dissolution of the GEM, the released primers mixed with the cell lysate and Master Mix, enabling the generation of barcoded, full-length cDNA from poly-adenylated mRNA after incubation. Silane magnetic beads were then used to remove excess biochemical reagents from the post-GEM reaction mixture.

Next, full-length, barcoded cDNA underwent PCR amplification to generate sufficient material for library construction. Enzymatic fragmentation and size selection were performed to optimize the cDNA amplicon size before library construction. During library construction, P5 and P7 adapters, a sample index, and R2 (read 2 primer sequence) were added via end repair, A-tailing, adaptor ligation, and PCR. The final libraries contained the P5 and P7 primers required for Illumina bridge amplification. Paired-end sequencing was conducted on an Illumina NovaSeq 6000 instrument following Illumina protocols and 10x Genomics sequencing specifications. The 8 bp index was read during an additional sequencing cycle that automatically followed the completion of read 1. Data generated during sequencing runs were simultaneously transferred to the YCGA high-performance computing cluster. To monitor quality in real time, a positive control (a prepared bacteriophage Phi X library) provided by Illumina was spiked into every lane at a concentration of 0.3%. Signal intensities were converted to individual base calls during the run using the system’s Real-Time Analysis software. Base calls were transferred from the machine’s dedicated personal computer to the Yale high-performance computing cluster via a 1-gigabit network mount for downstream analysis.

### Statistics

#### Weighted mean and standard error of mean.

The weighted mean (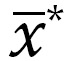) was calculated to equally represent male and female individuals in behavioral analyses using the following calculations: 


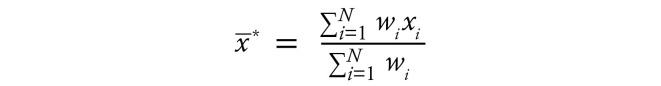


where *x_i_* is the observation, *w_i_* is the statistical weight (*w*_male_ = 1/*N*_male_ and *w*_female_ = 1/*N*_female_), *N* is the number of observations, and *M* is the number of weights. Weighted standard error of mean (SEM_w_) was calculated using the following calculations:


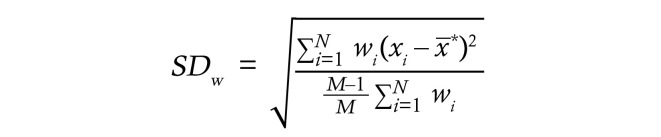


and


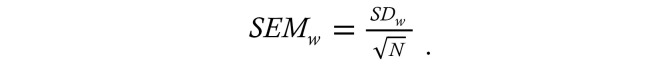


#### Statistical analysis.

Statistical analysis was performed using GraphPad Prism 9.0. A 2-tailed unpaired Student’s *t* test was used for comparisons between 2 groups, while 1-way ANOVA with Tukey’s multiple-comparison test was applied for analyses involving 3 or more groups with a single independent variable. Two-way ANOVA with Tukey’s multiple-comparison test was used when assessing the effects of 2 independent variables and their interaction (e.g., genotype and time point). The log-rank test with Bonferroni’s correction was used to compare survival across multiple groups. A significance threshold of α < 0.05 was applied.

#### Integration and clustering of snRNA-seq data.

Following alignment of sequencing reads to the pre-mRNA–containing mouse reference genomes (mm10) using the CellRanger count function (10x Genomics), ambient RNA was removed using CellBender (92). Doublets were identified using Scrublet (93) and removed. We followed standard single-cell RNA sequencing (scRNA-seq) analysis pipelines for pre-processing scRNA-seq count data (94). All snRNA-seq data were analyzed with Python (version 3.8.2), with pre-processing performed in SCANPY v1.6.0 (95). Briefly, genes expressed in fewer than 3 nuclei were removed. Nuclei that expressed < 500 genes or > 10% mitochondrial genes were excluded. The resulting UMI counts were normalized to library size and square-root-transformed. An approximate batch-balanced KNN (BBKNN) graph was employed for batch-effect correction, manifold learning, and clustering (96). For each cell, the 3 nearest neighboring cells in each condition were identified by Euclidean distance in a 100-dimensional principal component analysis space. This KNN graph was used as the basis for downstream analysis. Nuclei were clustered using the Leiden community detection method on the BBKNN graph with a resolution of 3. Preclusters of the same major cell type were merged when appropriate and manually annotated based on expression of previously described cell type–specific marker genes (33). Preclusters expressing marker genes of multiple cell types or lacking clear marker gene expression were removed.

#### Differential expression and GO analysis.

To address the inherent data sparsity in single-cell transcriptomics due to limited transcript capture (97), we applied Markov affinity-based graph imputation of cells method (98) within genotype before differential gene expression analysis to recover missing data, based on the BBKNN graph.

DEGs were calculated as previously described (33). Given the complexity and heterogeneity of gene expression patterns in single-cell transcriptomics, we used a combination of 3 metrics to determine DEGs: (a) the Wasserstein or EMD, (b) an adjusted *P* value from a 2-sided Mann-Whitney *U*/Wilcoxon rank-sum test with continuity correction and Benjamini-Hochberg adjustment, and (c) the binary logarithm of fold change between mean counts. The EMD, which represents the minimal cost required to transform 1 distribution to another, has been used to assess significant differences in gene expression between conditions (99, 100). Binary genotype comparisons were performed in each cell type for imputed expression. Genes with *P*_corrected_ < 0.01 and |EMD| ≥ 0.1 were considered significant. Gene set enrichment analyses of significantly DEGs were performed using over-representation analysis in GSEApy (101).

### Study approval

All animal procedures were conducted in compliance with the NIH *Guide for the Care and Use of Experimental Animals* (National Academies Press, 2011) and were approved by the Yale University IACUC.

### Data availability

All raw and processed data files from this study have been deposited into NCBI GEO under accession GSE319510. All data values in this work can be found in the Supporting Data Values file. The data and additional details on protocols used in this study are available from the corresponding author upon reasonable request. The scripts used in this study are accessible for academic use at the following link: https://github.com/ChrLeeee/OL-SCA1-cKI (commit ID 9160a03).

## Author contributions

CL, LT, and JL conceived and designed the study. CL and VO performed behavioral experiments. CL, RMG, EB, HR, and HK measured animal weight. RMG performed electron microscopy experiments, while CL, RMG, and HK analyzed the electron microscopy data. CL, RMG, EB, and HK conducted Western blot analyses. CL and EB performed immunohistochemistry. CL performed single-nucleus RNA sequencing and data analysis. AC regenerated SCA1 human and constitutive SCA1-KI mouse single-nucleus RNA-sequencing datasets using the updated pipeline. JPO provided SCA1 conditional KI animals. JL supervised this study and provided resources. CL drafted the manuscript, and all authors contributed to editing and provided comments.

## Conflict of interest

LT is a past employee/shareholder of Denali Therapeutics Inc. and a current full-time employee and shareholder of Pfizer, Inc.

## Funding support

This work is the result of NIH funding, in whole or in part, and is subject to the NIH Public Access Policy. Through acceptance of this federal funding, the NIH has been given a right to make the work publicly available in PubMed Central.

- NIH R01 AG066447, R01 AG074609, and R01 AG076154 to JL.
- NIH T32 NS007224 to LT.
- NIH/NIGMS Award 1S10OD030363-01A1 to Yale Center for Genomic Analysis.
- National Ataxia Foundation Graduate Research Fellowship to CL.
- Yale Wu Tsai Graduate Fellowship to RMG.

## Supplementary Material

Supplemental data

Unedited blot and gel images

Supplemental table 1

Supplemental table 2

Supplemental table 3

Supplemental table 4

Supplemental table 5

Supporting data values

## Version 1 05/11/2026

In-Press Preview

## Version 2 06/15/2026

Electronic publication
