# Supplementary material for: Oligodendrocyte dysfunction contributes to motor deficits and Purkinje cell axonopathy in spinocerebellar ataxia type 1
Source: J Clin Invest. 2026 May 7;136(12):e195723. doi: 10.1172/JCI195723 (PMC13262716; doi:10.1172/JCI195723)
Supplement: Supplemental data [file jci-136-195723-s033.pdf]

Supplemental Figure 1

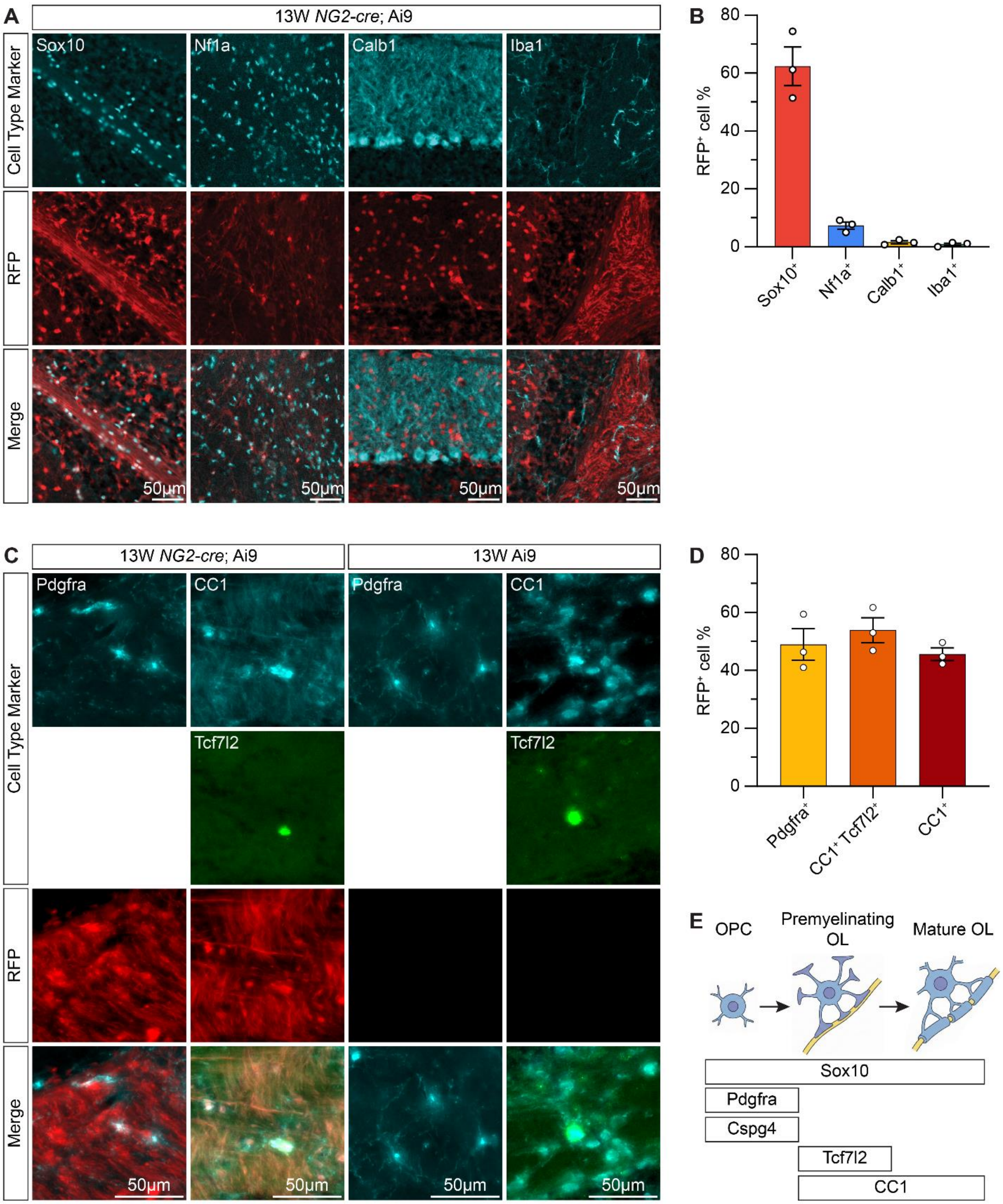

Supplemental Figure 1. Validation of Cre recombinase expression in OL lineage cells in *NG2-cre* mice

(A-D) IHC images (A and C) and quantifications (B and D) of 13-week-old *NG2-cre*; Ai9 reporter mice (n=3 animals). Markers used: Sox10 for oligodendroglia and myelin-rich white matter, Nf1a for astrocytes, Calb1 for PCs, and Iba1 for microglia (A and B). Pdgfra for OPCs, Tcf7l2 for premyelinating oligodendrocytes, and CC1 for oligodendrocytes (C and D). Scale bar: 50 μm.

(E) Schematic illustration of marker proteins used to identify oligodendroglia lineage cells, including Sox10, Pdgfra, Cspg4, Tcf7l2, and CC1.

Supplemental Figure 2

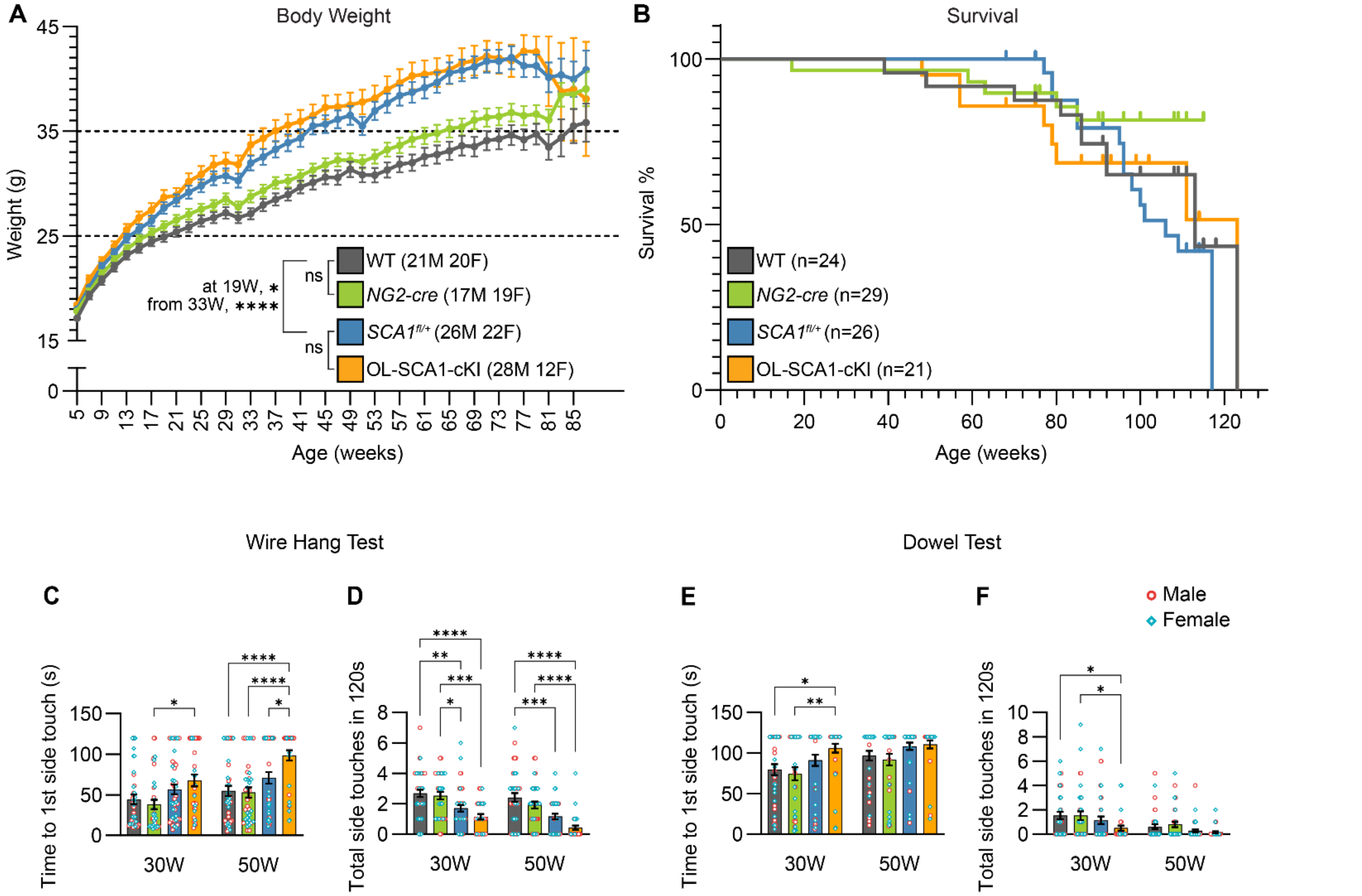

Supplemental Figure 2. Body weight, survival, and motor coordination test results in OL-SCA1-cKI mice

(A) Body weights of OL-SCA1-cKI and control mice, measured every two weeks starting at 5 weeks of age.

(B) Survival curves of OL-SCA1-cKI and control mice, showing no significant differences between genotypes (Mantel-Cox test).

(C-F) Motor coordination performance assessed using wire hang tests (C and D), dowel rod tests (E and F) in OL-SCA1-cKI and control mice at 30 and 50 weeks of age.

Two-way ANOVA with Tukey's multiple comparisons test was performed for (A and C-F). Data are presented as weighted mean ± SEM, equally representing male and female mice (A), or mean ± SEM (C-F). Statistical significance: **P*<0.05, ***P*<0.01, ****P*<0.001, *****P*<0.0001; ns, non-significant. The number of animals used for body weight measurements (A) and the survival study (B) is also indicated.

Supplemental Figure 3

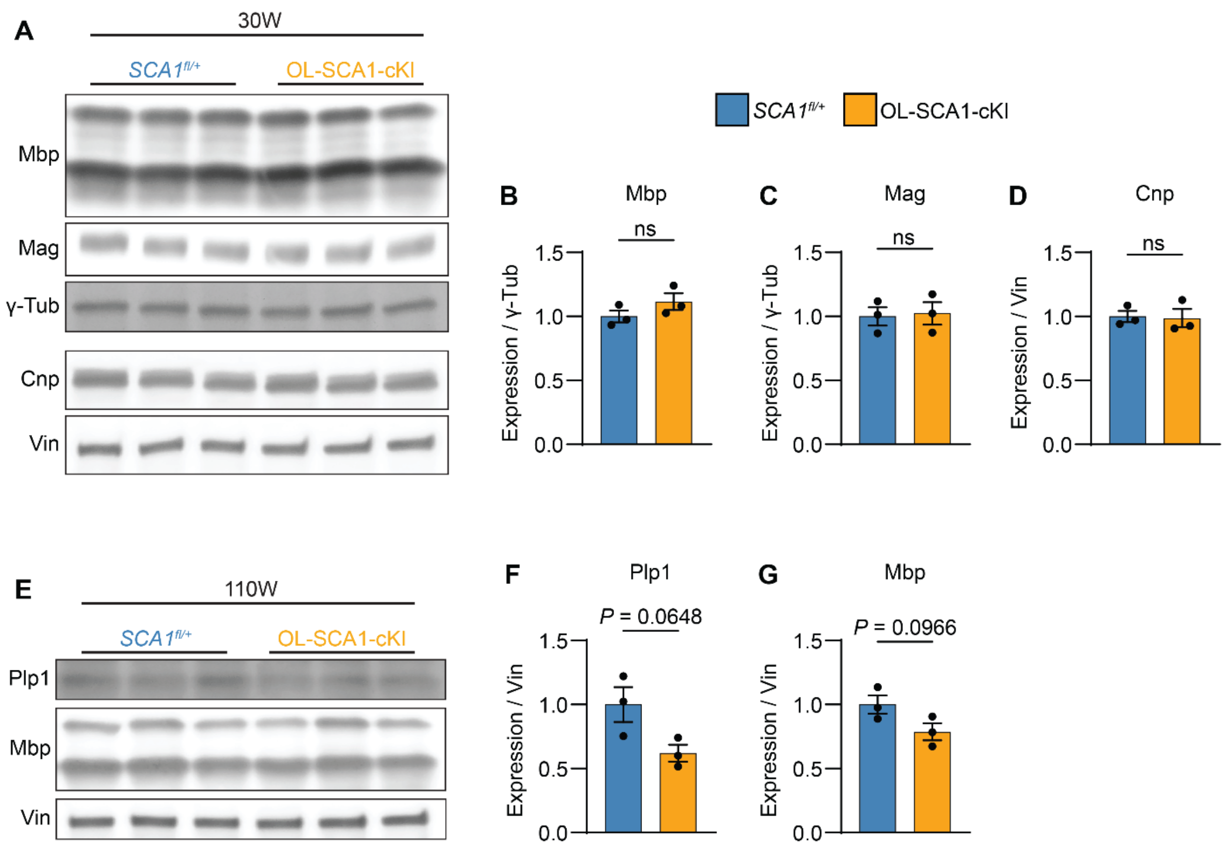

Supplemental Figure 3. Myelin protein expression in cerebella from OL-SCA1-cKI mice
(A-G) Western blot analysis of cerebellar myelin proteins at 30 weeks (A-D) and 110 weeks (E-G), with corresponding quantifications, showing a progressive trending reduction in myelin proteins at the bulk level. Data are presented as mean ± SEM, with n=3 mice per genotype. Statistical significance was determined using Student's *t*-tests. ns, non-significant.

Supplemental Figure 4

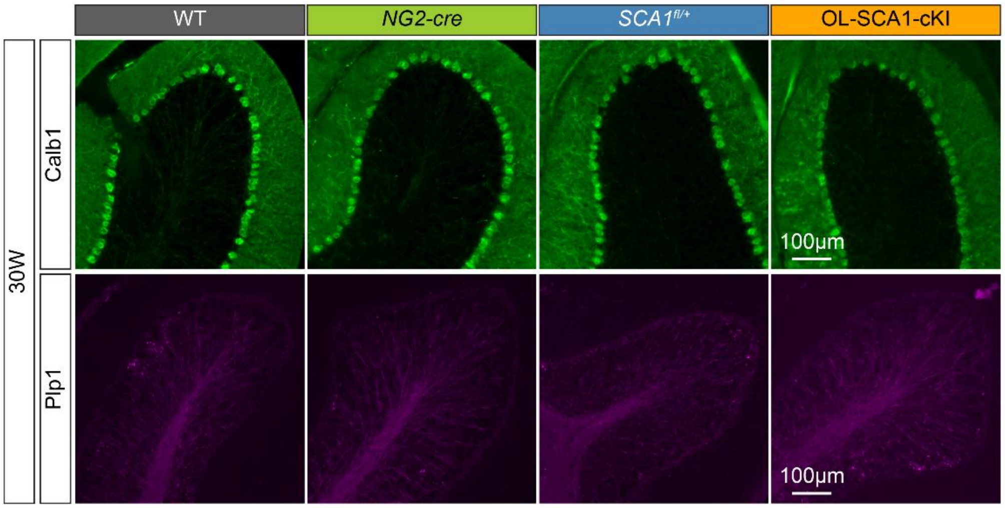

Supplemental Figure 4. Assessment of PC torpedoes and myelin debris at 30 weeks

IHC of cerebellar sections from OL-SCA1-cKI and control mice at 30 weeks, stained with Calb1 (top) and Plp1 (bottom). Scale bar: 100 µm (main images).

Supplemental Figure 5

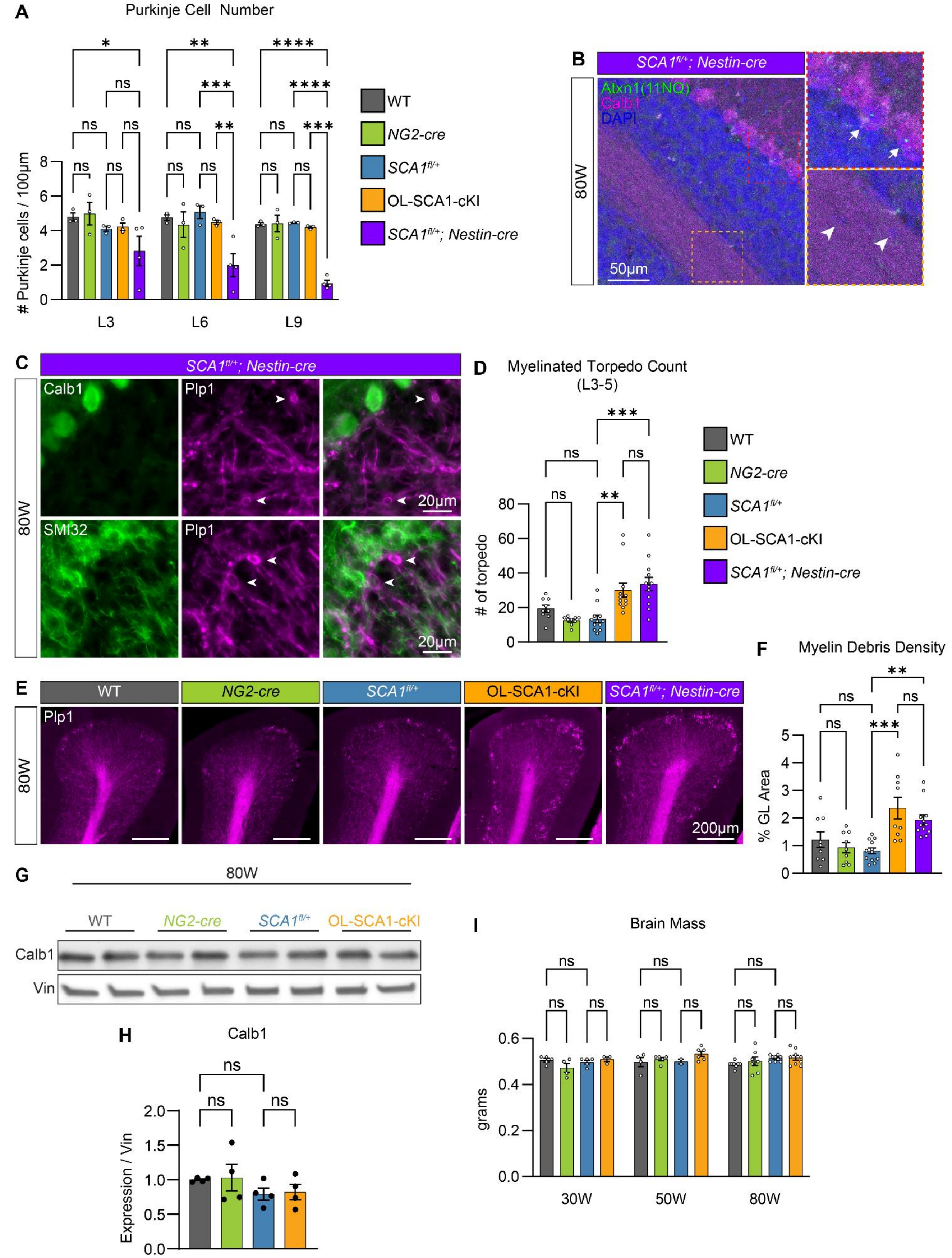

Supplemental Figure 5. PC number and calbindin-1 expression remain unchanged in OL-SCA1-cKI mice

(A) Quantification of PC numbers in cerebellar lobules (L) 3, 6, and 9 at 80 weeks. n=3-4 mice per genotype. Each data point represents an individual animal.

(B) IHC image of *SCA1$^{fl/+}$*; *Nestin-cre* mice at 80 weeks, stained for Atxn1(11NQ) and Calb1, showing intranuclear ataxin-1 inclusions in PCs but not in the white matter. Scale bar: 50 μm.

(C) Representative IHC images of *SCA1$^{fl/+}$*; *Nestin-cre* mice at 80 weeks, stained for Calb1 (top) or SMI32 (bottom), and co-stained with Plp1. Note that PC torpedoes (arrowheads) are Plp1^{+} but are negative for Calb1 or SMI32. Scale bar: 20 μm.

(D) Quantifications of Plp1^{+} myelinated PC torpedoes in the granular layer of cerebellar lobules 3-5 in OL-SCA1-cKI and control mice at 80 weeks. n=3-4 mice per genotype. Each data point represents an individual image measurement.

(E and F) Representative IHC images (E) and quantification (F) of myelin debris in the granular layer OL-SCA1-cKI and control mice at 80 weeks. n=3-4 mice per genotype. Data points represent individual measurements per image.

(G and H) Representative Western blot image (G) and quantification (H) of Calb1 protein expression levels in the cerebellum at 80 weeks, showing no statistically significant differences among genotypes. n=2 males and 2 females per genotype.

(I) Quantification of brain mass at 30, 50, and 80 weeks of age. Each data point represents an individual animal.

Data are presented as mean ± SEM. Statistical significance was determined using two-way ANOVA (A and I) or one-way ANOVA (D, F, H) with Tukey's multiple comparison analysis: *P<0.05, **P<0.01, ***P<0.001, ****P<0.0001; ns, non-significant.

Supplemental Figure 6

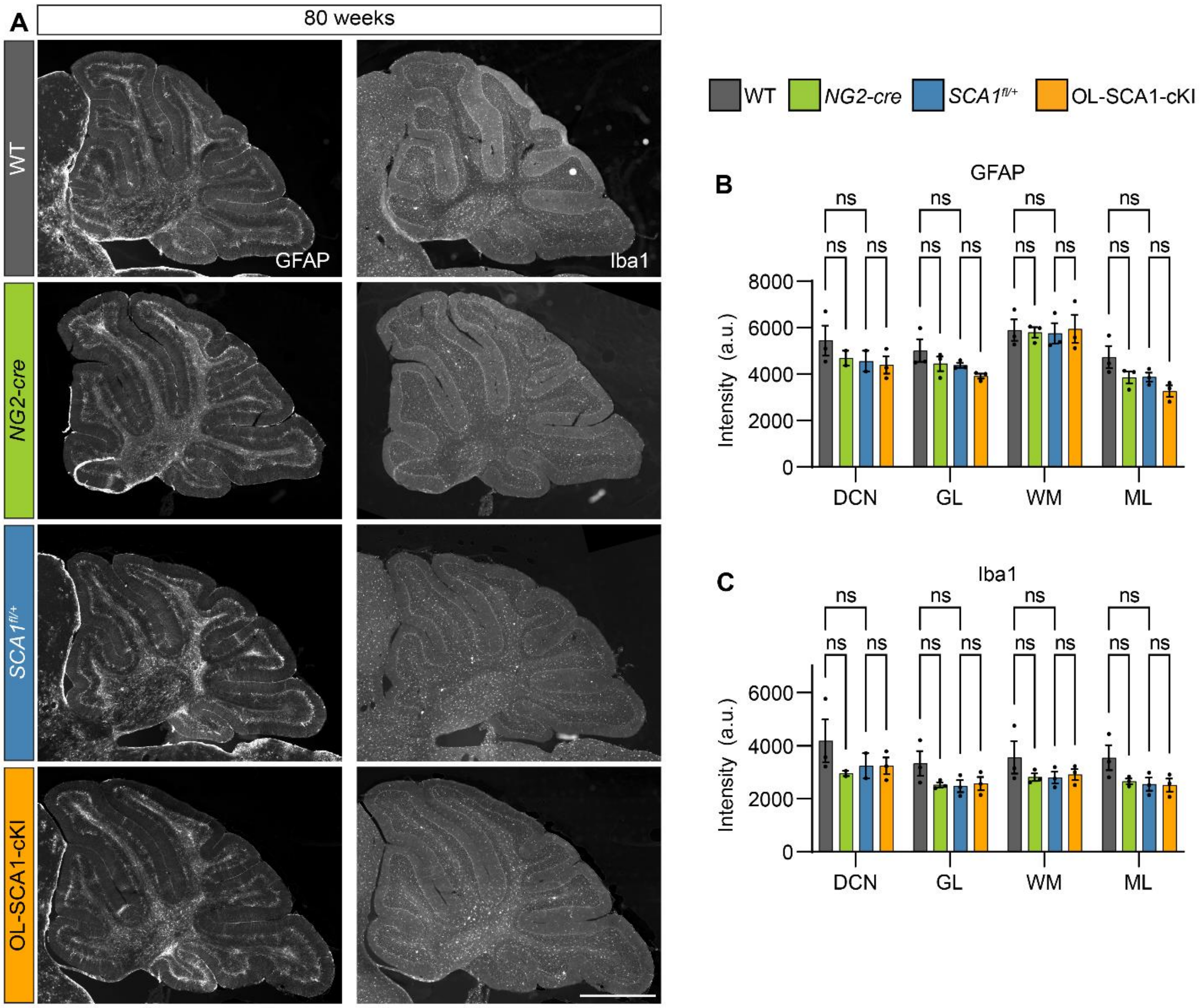

Supplemental Figure 6. No gliosis observed in OL-SCA1-cKI mouse cerebellum despite PC axon damage and myelin deficits

(A) IHC images of the cerebellar sections from OL-SCA1-cKI and control mice at 80 weeks, assessing astrogliosis (GFAP) and microgliosis (Iba1). Scale bar: 1 mm.

(B and C) Quantifications of GFAP fluorescence signal intensity (B) and Iba1 fluorescence signal intensity (C) in the deep cerebellar nuclei (DCN), granular layer (GL), white matter (WM), and molecular layer (ML). Data are presented as mean ± SEM (n=3 mice per genotype), with each data point representing an individual animal. Statistical analysis was performed using two-way ANOVA with Tukey's multiple comparison analysis, showing no significant differences between the selected comparisons. ns, non-significant.

Supplemental Figure 7

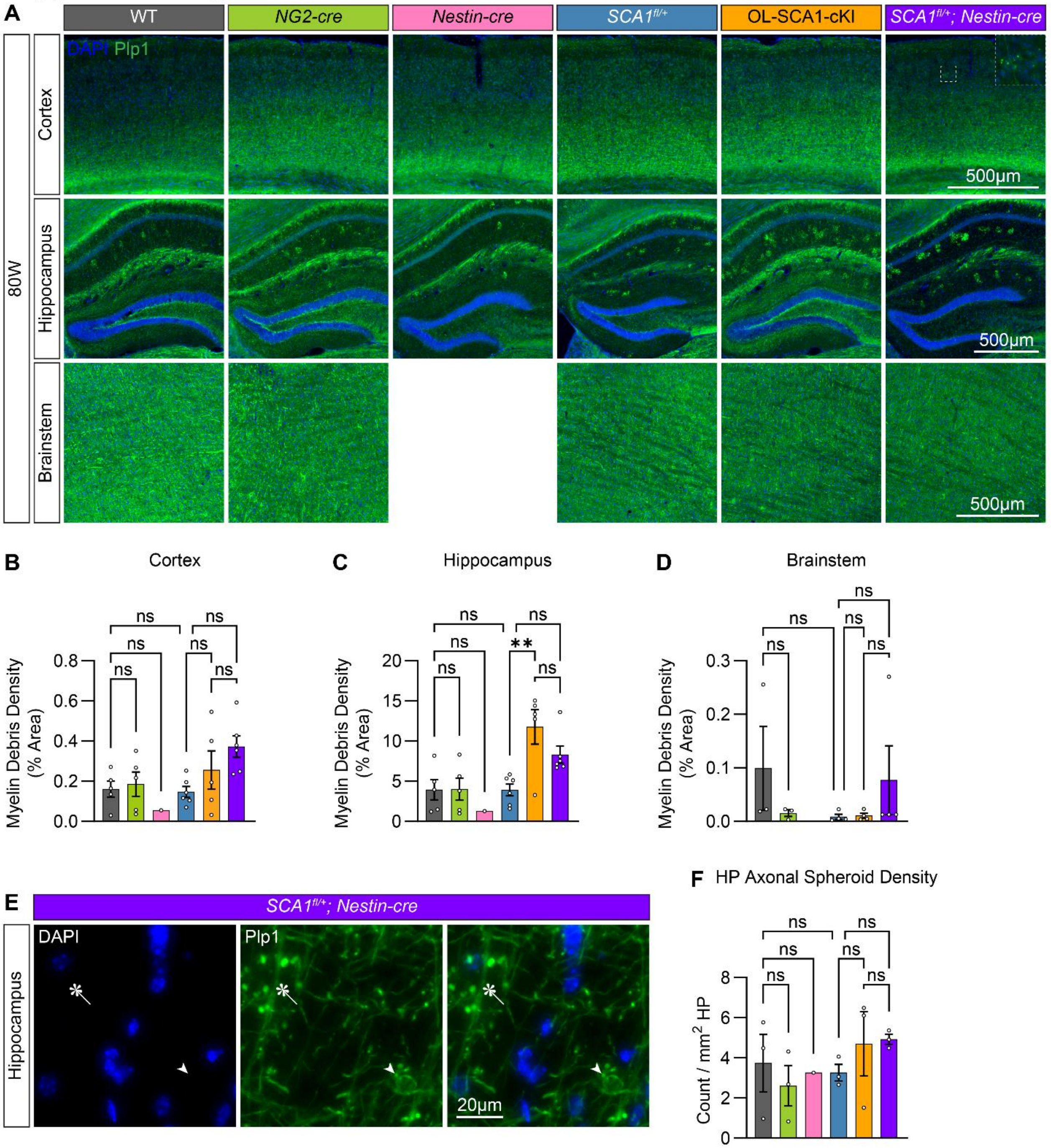

Supplemental Figure 7. Assessment of myelin debris and axonal swelling in extracerebellar regions in OL-SCA1-cKI mice

(A) IHC images of the cortex (top), hippocampus (middle), and brainstem (bottom) from OL-SCA1-cKI and control mice at 80 weeks. Scale bar: 500 μm.

(B-D) Quantifications of $Plp1^+$ myelin debris in the cortex (B), hippocampus (C), and brainstem (D). Data are presented as mean ± SEM. n=3-6 mice per genotype, except for *Nestin-cre* (n=1 mouse in the cortex and hippocampus). Data points represent individual animals. Statistical significance was determined using one-way ANOVA with Tukey's multiple comparison test: $^{**}P<0.01$; ns, non-significant.

(E) Representative enlarged IHC image of the hippocampus from a $\mathit{SCA1}^{fl/+}$; *Nestin-cre* mouse. Asterisk-headed arrow indicates myelin debris; arrowhead indicates $Plp1^+$ $DAPI^-$ myelinated axonal spheroid. Scale bar: 20 μm.

(F) Quantification of myelinated axonal spheroids in the hippocampus. n=3 mice per genotype, except for *Nestin-cre* (n=1). Data points represent individual animals. Statistical significance was determined using one-way ANOVA with Tukey's multiple comparison test: All comparisons are non-significant (ns).

Supplemental Figure 8

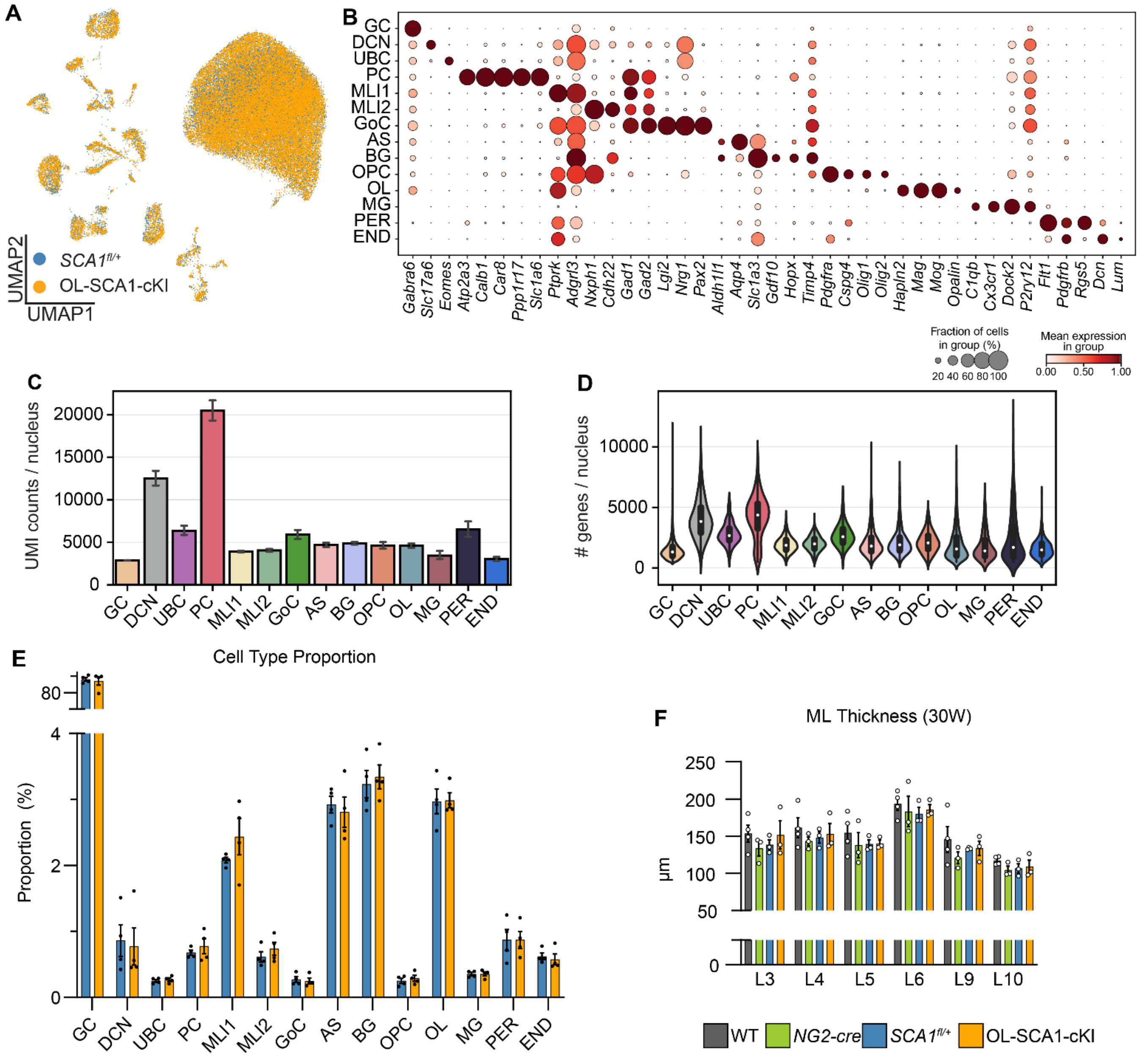

Supplemental Figure 8. snRNA-seq analysis and PC pathology assessment of the cerebellum in OL-SCA1-cKI mice

(A) UMAP plot of cerebellar cells from OL-SCA1-cKI and $SCA1^{fl/+}$ littermate control mice at 30 weeks, colored by genotype. Sample sizes: $SCA1^{fl/+}$, n=28,908; OL-SCA1-cKI, n=35,396 nuclei.

(B) Dot plots displaying marker gene expression across cerebellar cell types.

(C) Bar plots showing the number of unique molecular identifiers (UMIs) detected per cell for each cell type. Data are presented as means with 95% confidence intervals.

(D) Violin plots illustrating the number of genes detected per cell in each cell type before imputation with MAGIC. The median (white dot) and interquartile range are shown.

(E) Bar graphs depicting cell type proportions. Data points represent individual animals, presented as mean ± SEM. Statistical analysis was performed using two-way ANOVA with Tukey's multiple comparison analysis, revealing no significant differences between genotypes.

Abbreviations: GC= granule cell, DCN= deep cerebellar nuclei neuron, UBC= unipolar brush cell, PC= Purkinje cell, MLI1= molecular layer interneuron 1, MLI2= molecular layer interneuron 2, GoC= Golgi cell, AS= astrocyte, BG= Bergmann glia, OPC= oligodendrocyte progenitor cell, OL= oligodendrocyte, MG= microglia, PER= pericyte, END= endothelial cell.

(**F**) Quantification of molecular layer (ML) thickness in anterior (L3 and L4), medial (L5 and L6), and posterior (L9 and L10) cerebellar regions in 30-week-old OL-SCA1-cKI and control mice. Data points represent individual animals, with n=3 mice per genotype. Data are presented as mean ± SEM. Statistical analysis was performed using two-way ANOVA with Tukey's multiple comparison test: All comparisons are non-significant (ns).

Supplemental Figure 9

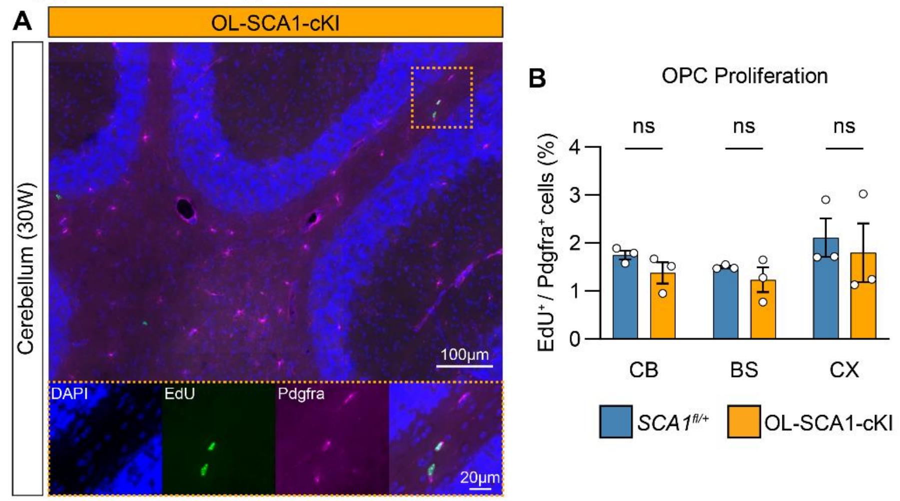

Supplemental Figure 9. OPC proliferation is not altered in OL-SCA1-cKI mice

(A) Representative IHC image of the cerebellum from a OL-SCA1-cKI mouse at 30 weeks of age, following 3 consecutive days of IP injection with 5-ethynyl 2'-deoxyuridine (EdU). Scale bar: 100 μm (main image) and 20 μm (enlarged images).

(B) Quantification of EdU^+ proliferating cells among $Pdgfra^+$ OPCs in the cerebellum (CB), brainstem (BS), and cortex (CX) of OL-SCA1-cKI and $\textit{SCA1}^{fl/+}$ littermate control mice (n=3 mice per genotype). Data points represent individual animals, presented as mean ± SEM. Statistical analysis was performed using two-way ANOVA with Sidak's multiple comparison analysis, showing no significant differences between genotypes.

Supplemental Figure 10

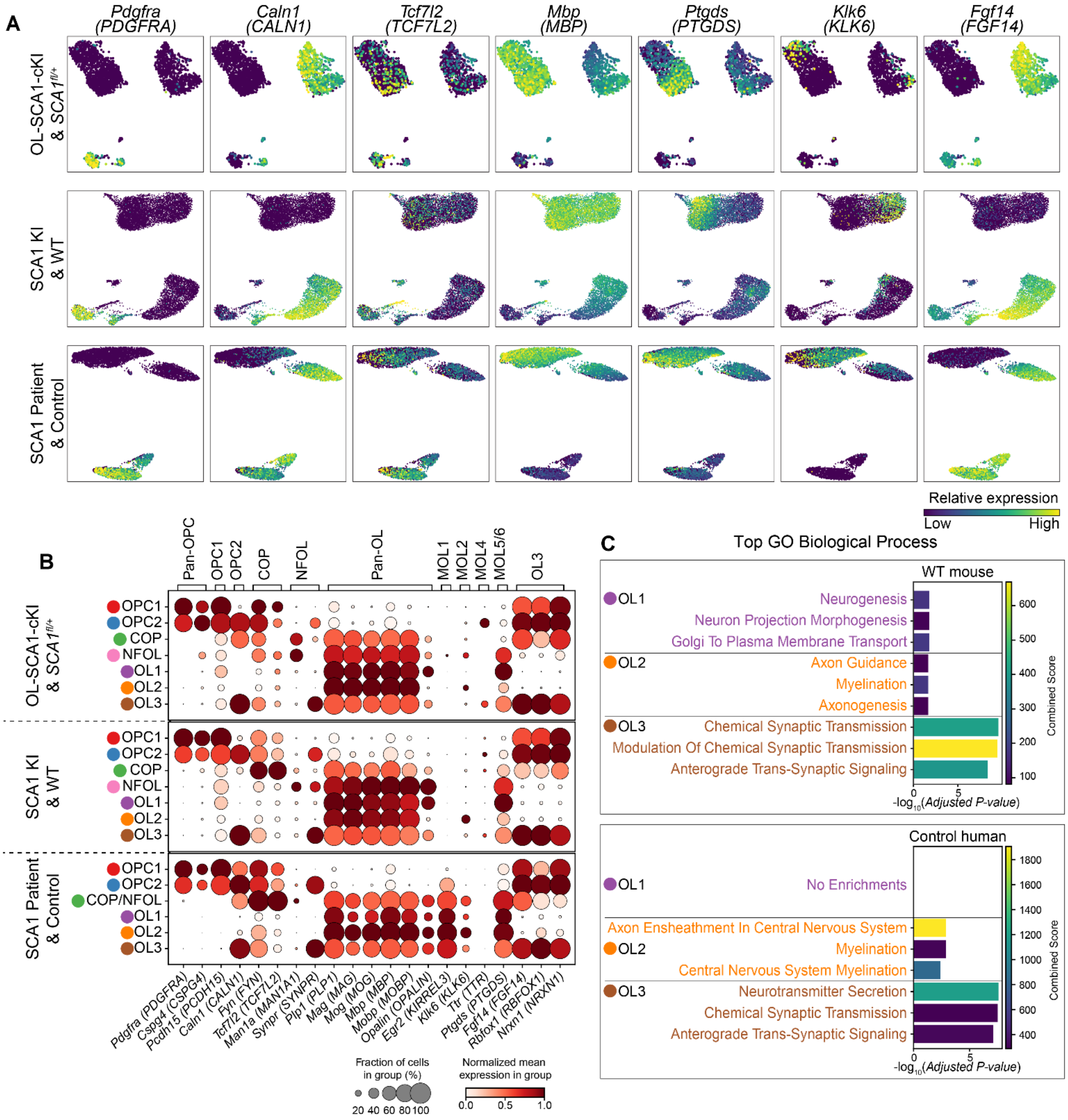

Supplemental Figure 10. Subclustering analysis reveals common cerebellar oligodendroglia subtypes and highlights their distinct functional roles

(A) UMAP plots illustrating the relative expression of key marker genes across oligodendroglial subtypes in OL-SCA1-cKI (top), constitutive SCA1 KI (middle), and human SCA1 (bottom) datasets.

(B) Dot plots showing marker gene expression across identified oligodendroglia subtypes. Dot size represents the proportion of cells expressing the gene, while color intensity reflects average expression levels.

(C) Top 3 significantly enriched GO biological processes of the top 100 genes of OL1, OL2, and OL3 in wild-type mouse (top) and control human (bottom) samples.

Supplemental Figure 11

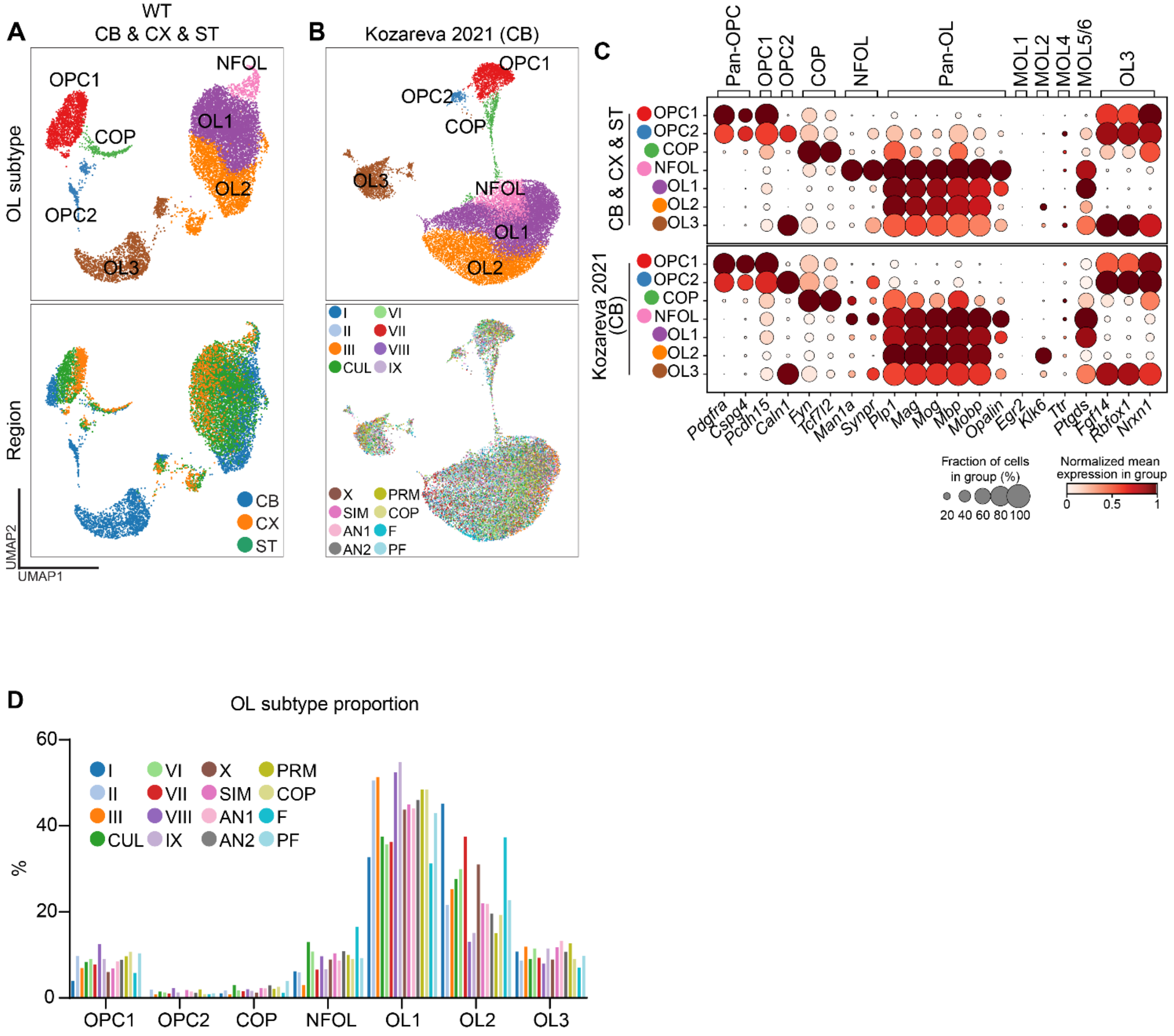

Supplemental Figure 11. OL3 is a cerebellum-specific OL subtype, while all three OL subtypes exhibit a uniform distribution across cerebellum subregions

(A) UMAP plots of oligodendroglia from cross-region datasets, including cerebellum (CB), cortex (CX), and striatum (ST). The top plot is colored by annotated oligodendroglia subtypes, while the bottom plot is colored by brain region.

(B) UMAP plots of oligodendroglia from cross-cerebellar subregion dataset(69), encompassing lobules (I, II, III, VI, VII, VIII, IX, X), ansiform lobule (AN), copula pyramidis (COP), culmen (CUL), flocculus (F), paraflocculus (PF), paramedian lobule (PRM), and simple lobule (SIM). The top plot is colored by oligodendroglia subtypes, while the bottom plot is colored by cerebellar subregions.

(C) Dot plots displaying marker gene expression across oligodendroglia subtypes, with dot size representing the proportion of cells expressing the gene and color intensity indicating average expression levels.

(D) Bar plots showing the distribution of OL subtypes across cerebellar subregions.

Supplemental Figure 12

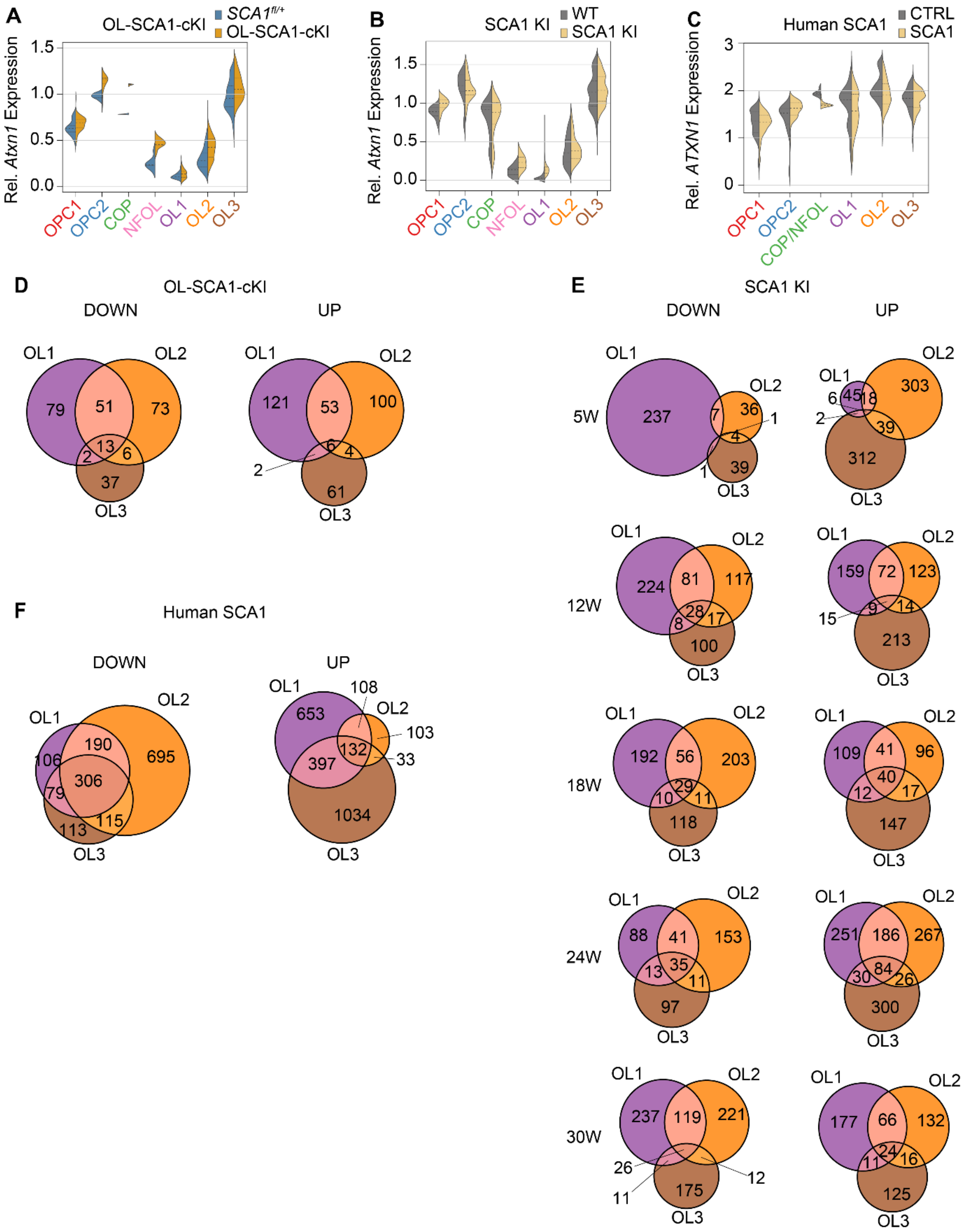

Supplemental Figure 12. Differential gene dysregulation among OL subtypes in SCA1

(A-C) Violin plots showing relative expression levels of mouse *Atxn1* (or human *ATXN1*) across oligodendroglia subtypes in the OL-SCA1-cKI (A), 18-week-old SCA1 KI (B), and human SCA1 (C) cerebellar datasets.

(D-F) Venn diagrams illustrating the overlap of down- (left) and up-regulated (right) DEGs among mature OL subtypes (OL1, OL2, OL3) in the OL-SCA1-cKI (D), constitutive SCA1 KI (E), and human SCA1 (F) datasets. The numbers within the diagrams indicate shared and unique DEGs across subtypes, highlighting both conserved and subtype-specific gene dysregulation patterns in SCA1.

Supplemental Figure 13

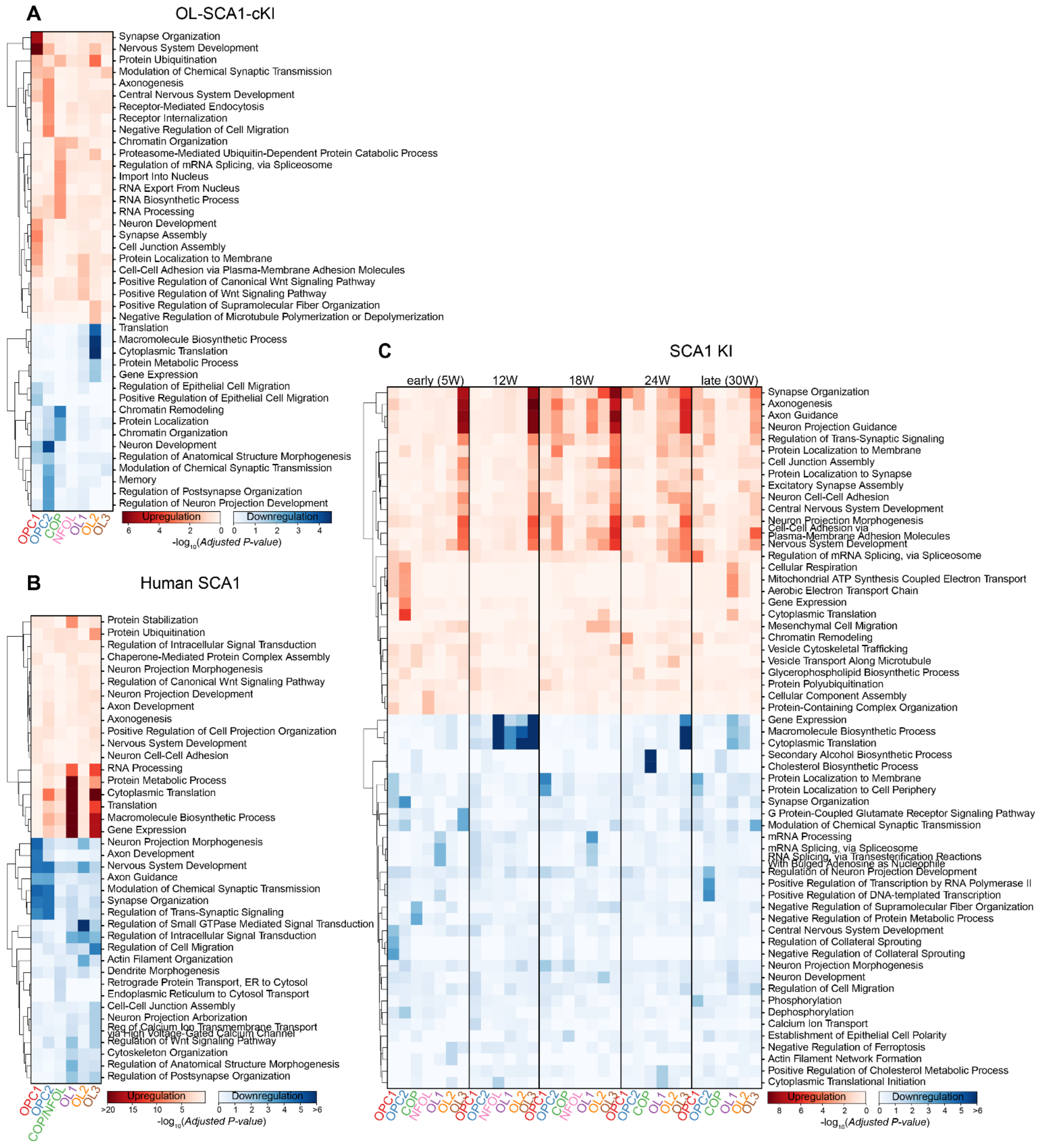

Supplemental Figure 13. GO analyses across SCA1 oligodendroglia subtypes

(A-C) Heatmaps showing the top GO terms associated with up- and down-regulated DEGs across all oligodendroglia subtypes in the OL-SCA1-cKI (A), human SCA1 (B), and SCA1 KI (C) datasets.

Supplemental Figure 14

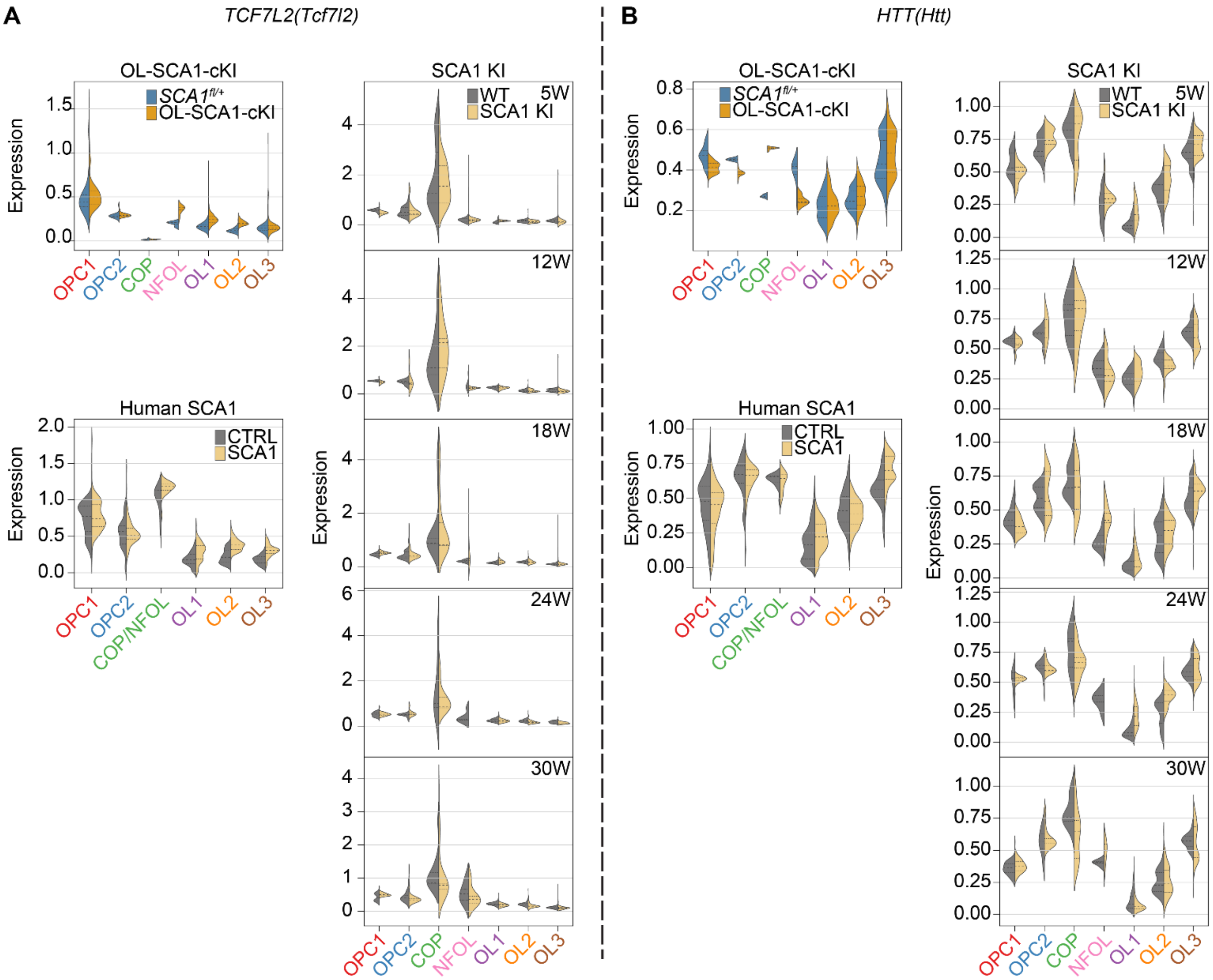

Supplemental Figure 14. Expression patterns of *TCF7L2* and *HTT* across SCA1 oligodendroglia subtypes

(A-B) Violin plots showing relative expression levels of *TCF7L2*(*Tcf7l2*) (A) and *HTT*(*Htt*) (B) across OL subtypes from the OL-SCA1-cKI (upper left), human SCA1 (lower left), and SCA1 KI (right) datasets.
