# Supplementary figures and images for: Oligodendrocyte dysfunction contributes to motor deficits and Purkinje cell axonopathy in spinocerebellar ataxia type 1
Source: J Clin Invest. 2026 May 7;136(12):e195723. doi: 10.1172/JCI195723 (PMC13262716; doi:10.1172/JCI195723)

Supp Fig 3A

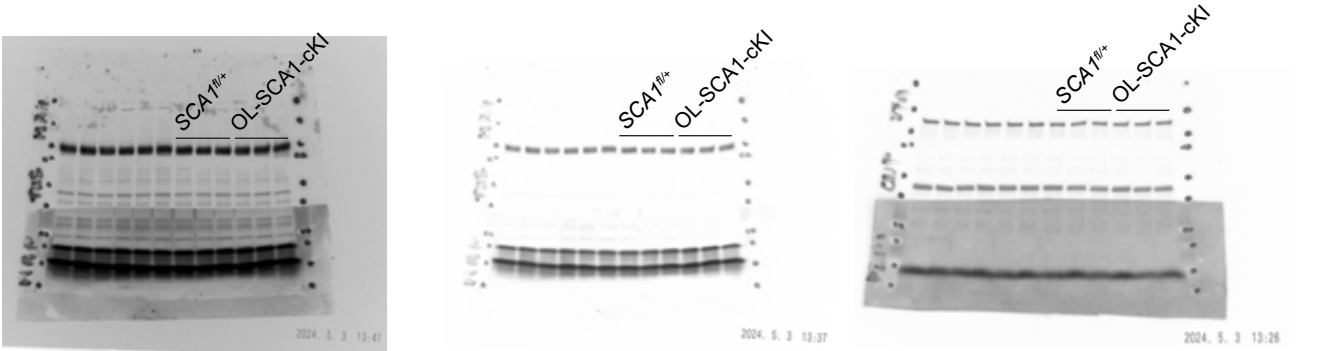

Supp Fig 3E

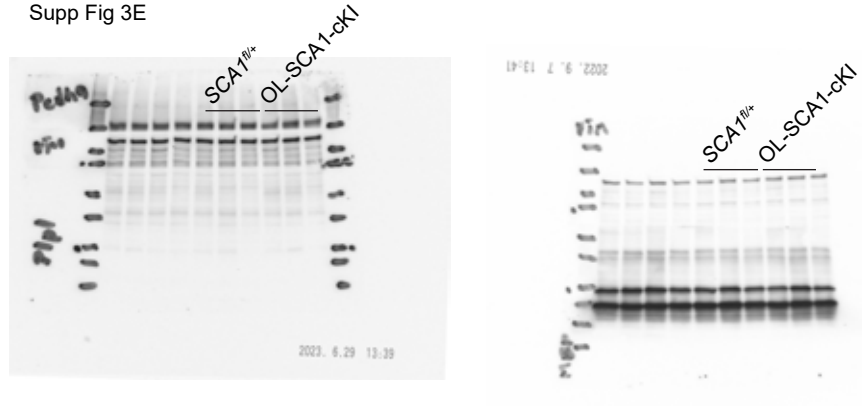

Supp Fig 4F

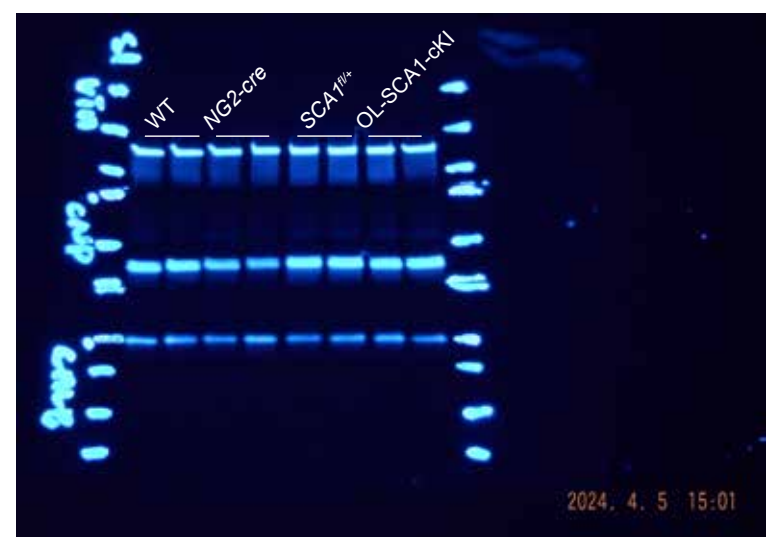

Supplement: Unedited blot and gel images [file jci-136-195723-s034.pdf]
